# In vitro cellular reprogramming to model gonad development and its disorders

**DOI:** 10.1126/sciadv.abn9793

**Published:** 2023-01-04

**Authors:** Nitzan Gonen, Caroline Eozenou, Richard Mitter, Maëva Elzaiat, Isabelle Stévant, Rona Aviram, Andreia Sofia Bernardo, Almira Chervova, Somboon Wankanit, Emmanuel Frachon, Pierre-Henri Commère, Sylvie Brailly-Tabard, Léo Valon, Laura Barrio Cano, Romain Levayer, Inas Mazen, Samy Gobaa, James C. Smith, Kenneth McElreavey, Robin Lovell-Badge, Anu Bashamboo

**Affiliations:** ^1^ The Mina and Everard Goodman Faculty of Life Sciences and the Institute of Nanotechnology and Advanced Materials, Bar-Ilan University, Ramat Gan 5290002, Israel.; ^2^ The Francis Crick Institute, 1 Midland Road, London NW1 1AT, UK.; ^3^ Institut Pasteur, Université de Paris, CNRS UMR3738, Human Developmental Genetics, F-75015 Paris, France.; ^4^ Bioinformatics Core, The Francis Crick Institute, 1 Midland Road, London NW1 1AT, UK.; ^5^ National Heart and Lung Institute, Imperial College London, London, UK.; ^6^ Department of Stem Cell and Developmental Biology, Institut Pasteur, Paris 75724, France.; ^7^ Biomaterials and Microfluidics Core Facility, Institut Pasteur, F-75015 Paris, France.; ^8^ Cytometry and Biomarkers, Centre de Ressources et Recherches Technologiques (C2RT), Institut Pasteur, F-75015 Paris, France.; ^9^ Assistance Publique-Hôpitaux de Paris, Bicêtre Hospital, Molecular Genetics, Pharmacogenetics, and Hormonology, Le Kremlin-Bicêtre, France.; ^10^ Institut Pasteur, Université de Paris, CNRS UMR3738, Cell Death and Epithelial Homeostasis, F-75015 Paris, France.; ^11^ Genetics Department, National Research Center, Cairo, Egypt.

## Abstract

During embryonic development, mutually antagonistic signaling cascades determine gonadal fate toward a testicular or ovarian identity. Errors in this process result in disorders of sex development (DSDs), characterized by discordance between chromosomal, gonadal, and anatomical sex. The absence of an appropriate, accessible in vitro system is a major obstacle in understanding mechanisms of sex-determination/DSDs. Here, we describe protocols for differentiation of mouse and human pluripotent cells toward gonadal progenitors. Transcriptomic analysis reveals that the in vitro–derived murine gonadal cells are equivalent to embryonic day 11.5 in vivo progenitors. Using similar conditions, Sertoli-like cells derived from 46,XY human induced pluripotent stem cells (hiPSCs) exhibit sustained expression of testis-specific genes, secrete anti-Müllerian hormone, migrate, and form tubular structures. Cells derived from 46,XY DSD female hiPSCs, carrying an *NR5A1* variant, show aberrant gene expression and absence of tubule formation. CRISPR-Cas9–mediated variant correction rescued the phenotype. This is a robust tool to understand mechanisms of sex determination and model DSDs.

## Teaser

Pluripotent cell-derived in vitro system is established to model mammalian gonad development and disorders of sex development.

## INTRODUCTION

During embryonic gonad development, the bipotential genital ridge adopts either a testicular or an ovarian cell fate. In the mouse, at around embryonic day 10.0 (E10.0), somatic cells migrating from the coelomic epithelium overlaying the mesonephros, as well as from the latter, give rise to the bipotential gonad. Several key factors are expressed in the genital ridge at this stage including *Wt1* (Wilms’ tumor 1) (*1*, *2*), *Sf1/Nr5a1* (steroidogenic factor 1) (*3*, *4*), *Gata4* (GATA binding protein 4) (*5*–*7*), *Cbx2* (chromobox protein homolog 2/M33) (*8*, *9*), *Zfpm2*/*Fog2* (friend of GATA protein 2) (*7*), *Lhx9* (Lim homeobox 9) (*10*), and *Emx2* (empty spiracles homeobox 2) (*11*, *12*). The developing gonad begins to become sexually dimorphic at the molecular level at around E10.7 (*13*–*16*) with the expression of *Sry* (Sex-determining region on Y) in the supporting cell precursors, leading to their differentiation into Sertoli cells (*17*–*19*). Sertoli cells then orchestrate testis cord formation and the differentiation of other somatic testicular lineages and the gonocytes. SRY up-regulates the expression of *Sox9*, a key transcription factor (TF), which, beyond a critical threshold, is both necessary and sufficient to induce and maintain Sertoli cells (*20*–*26*). *Sox9*, along with other pro-testis factors (*Gata4*, *Wt1*, *Sf1*, *Dmrt1*, and *Fgf9*), constitute a gene regulatory network that not only guides the precursor cells toward a testis fate but also opposes the network required for the formation of ovarian cell types (*19*, *27*, *28*).

Ovarian development requires *Rspo1*/*Wnt4*/β*-Catenin*, *Foxl2*, and the recently identified *Runx1* signaling networks (*29*). The RSPO1/WNT4 signaling pathway stabilizes β*-catenin*, which counteracts the establishment of a pro-testis *Sox9*/*Fgf9* network (*30*–*33*). In mice, FOXL2 is required to maintain ovarian identity postnatally, because the ablation of *Foxl2* in the murine adult ovary leads to trans-differentiation of granulosa cells into Sertoli cells (*34*). *Runx1* has complementary/redundant roles with *Foxl2* to maintain fetal granulosa cell identity and combined loss of *Runx1* and *Foxl2* results in masculinization of fetal ovaries (*29*).

Our understanding of sex-determination stems from embryological, genetic, and transcriptomic studies in mice and genetic analysis of humans with errors of sex determination. These individuals present with a phenotypic spectrum of conditions termed disorders/differences of sex development (DSDs), defined as congenital conditions in which development of chromosomal, gonadal, and anatomical sex is discordant (*35*). However, a major bottleneck in further understanding mouse, but especially human sex determination, is the lack of a robust in vitro model that accurately recapitulates in vivo gonad formation and development. Mouse models of variants associated with DSDs are limited by not only the expense and labor-intensive nature of producing mutant mouse lines (*35*, *36*) but also insufficient evolutionary conservation in sex determination between mice and humans (*37*–*39*). Primary gonadal cells do not survive for long, and their gene expression patterns rapidly diverge from normal under standard in vitro culture conditions (*40*). Although several Sertoli and granulosa cell lines have been established, these differ in expression profiles compared to their in vivo counterparts, limiting their usefulness (*41*). Recently, several attempts have been made to generate Sertoli and Leydig cells in vitro starting from pluripotent/multipotent cells, by either introducing exogenous TFs or using sequential supplement-enriched media. Direct reprogramming of fibroblasts has also been attempted. However, the resultant populations lack appropriate and sustained expression of key testis-specific markers (*40*, *42*–*48*). Except in the report by Yoshino *et al.* (*48*), no attempts were made to assess the entire transcription profiles, make comparisons to in vivo counterparts, or determine the functional ability of the in vitro–derived gonadal cells. Moreover, none of these studies attempted to model DSDs.

Here, we developed a robust protocol for sequential differentiation of mouse embryonic stem cells (mESCs) toward gonadal progenitors using defined medium. Analysis of the transcriptome by RNA sequencing (RNA-seq) shows that these in vitro–derived cells are highly similar to progenitors of E11.5 mouse gonads, which was further confirmed by comparison with published single-cell RNA-seq (scRNA-seq) data of murine embryonic gonads (*15*, *16*). Using the conditions optimized on mESCs, we derived Sertoli-like cells from human induced pluripotent stem cells (hiPSCs) derived from a healthy 46,XY male. The in vitro differentiated population shows sustained expression of testis-specific genes, secrete anti-Müllerian hormone (AMH), migrate, and spontaneously aggregate. Sorted cells can organize into three-dimensional (3D) tubular structures on a specially designed microfluidic chip. Last, we also show that this protocol can be successfully used to model aspects of gonadal development with a naturally occurring human sex-reversing gene variant in vitro*.*

## RESULTS

### Deriving TESCO-CFP;R26-rtTA XY mESCs

**Fig. 1.**
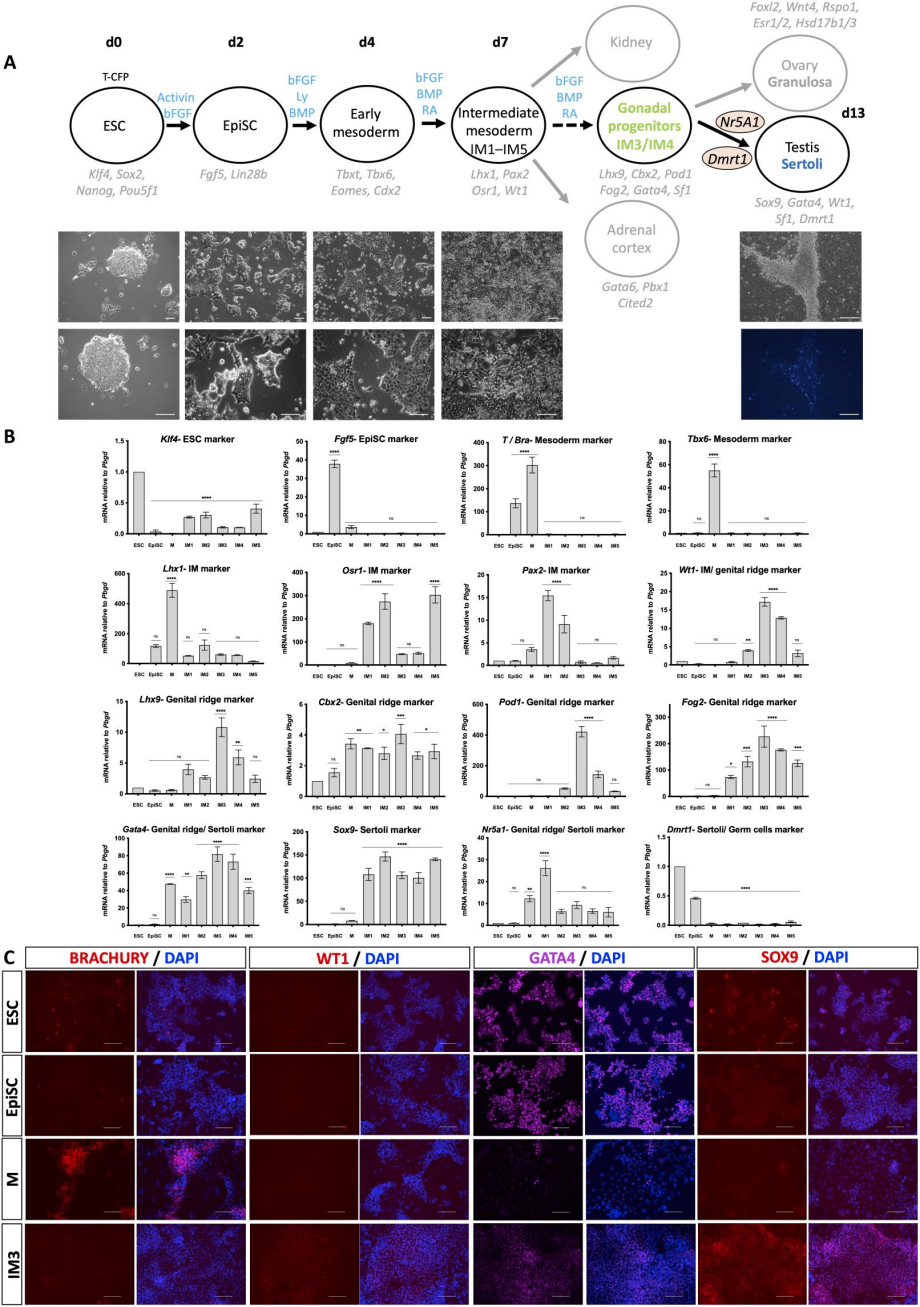
Differentiation of mouse ESCs toward gonadal cells. (**A**) Schematic representation of the differentiation protocol. T-CFP ESC (T-CFP;R26-rtTA, XY mESCs) were differentiated to EpiSCs followed by mesoderm induction, IM differentiation, and gonadal progenitor specification. Forced expression of *Sf1* and *Dmrt1* induced Sertoli-like cells expressing CFP, which create tubule-like structures. Growth factors added at each step are highlighted in blue above the arrows. Key markers of each stage are labeled in light gray. The time scale of the differentiation is presented at the top (day 0 to day 13). Representative bright-field (BF) and fluorescent images are depicted below the schematic representation. Scale bars, 100 μm. d0, day 0. (**B**) qPCR analysis of cells undergoing differentiation. Gene names are presented in the title. ESC, embryonic stem cells; EpiSC, epiblast stem cells; M, mesoderm; IM, intermediate mesoderm. Data are presented as mean 2^−ΔΔ*C*t^ values normalized to the housekeeping gene *Pbgd*. Error bars show SEM of 2^−ΔΔ*C*t^ values. Statistical analysis was performed using one-way analysis of variance (ANOVA) on the 2^−ΔΔCt^ values (*P < 0.05, **P < 0.01, ***P < 0.001, and ****P < 0.0001). ns, not significant. (**C**) Immunostaining of cells undergoing differentiation (ESC, EpiSC, M, and IM3). Staining was done using the BRACHURY (red), WT1 (red), GATA4 (magenta), and SOX9 (red) antibodies. The stained protein is depicted at the top bar. Left of each bar is the protein staining alone, and right is merge with 4′,6-diamidino-2-phenylindole (DAPI). Scale bars, 100 μm.

To enable differentiation of mESCs toward gonadal progenitors, we developed protocols that mimic the known pathway of gonad development (Fig. 1A), whereby pluripotent cells differentiate toward early mesoderm progenitors followed by further regional specification toward the mesoderm from which the gonads originate, which includes intermediate mesoderm (IM) and associated coelomic epithelium.

To facilitate the screening of culture conditions, we first derived ESCs from mice carrying the Sertoli cell–specific TES Core-CFP (TESCO-CFP) reporter (*49*). However, we also included the Rosa26-rtTA allele, which permits the forced expression, if required, of critical TFs using the doxycycline-inducible system. Experiments were performed using a clone, which was XY, TESCO-CFP;R26-rtTA.

### Differentiating mESC toward gonadal progenitors

Differentiation of mESCs was initiated by passaging them in media containing 20% knockout serum replacement (KSR). Cells were then differentiated to an epiblast stem cell (EpiSC)–like state by culture in N2B27 media supplemented with activin (20 ng/ml), basic fibroblast growth factor (bFGF; 12 ng/ml), and 1% KSR for 2.5 days as described by Hayashi *et al.* (*50*), who used a similar approach to generate primordial germ cell–like cells (PGCLCs) from mESCs. Quantitative polymerase chain reaction (qPCR) analysis showed that *Klf4* (Kruppel like factor 4) was highly expressed in ESCs and reduced in EpiSCs, while *Fgf5* (fibroblast growth factor 5) was induced in EpiSCs, suggesting that EpiSCs were successfully generated (Fig. 1B).

EpiSCs were then differentiated into early mesoderm progenitors by exposing the cells to a chemically defined media supplemented with bFGF (20 ng/ml), 10 μM LY294002, and bone morphogenetic protein 4 (BMP4) (10 ng/ml; termed FLyB) for 36 hours, on the basis of a protocol developed by Bernardo *et al.* (*51*). Robust mesodermal differentiation was evident by morphological changes in the cells (Fig. 1A), qPCR analysis, which showed high induction of *T* (Brachyury, *Tbxt*-T-box TF T) and *Tbx6* (T-box TF 6) expression (Fig. 1B), and immunostaining with an antibody against BRACHYURY, which showed high levels of BRA protein expression (Fig. 1C). Relying on the BMP4 gradient to which cells in the region of the IM cells are exposed, we tested five different conditions (termed IM1 to IM5) and screened the cells by marker analysis to identify the optimal protocol to obtain regional differentiation of the mesodermal cells to those most likely to be competent to form the gonads. All conditions used a defined media supplemented with bFGF (5 ng/ml) and medium levels of BMP4 (20 ng/ml). We then added additional factors including Retinoic Acid (RA, 100 nM), Activin A (10 ng/ml), and CHIR99021 (WNT agonist, 0.3 μM) (described in Materials and Methods). IM induction was performed for 3.5 days after which RNA was extracted and qPCR analysis was performed for four major IM markers including *Lhx1* (Lim homeobox 1), *Osr1* (odd skipped related TF 1), *Pax2* (paired box gene 2), and *Wt1* (Wilms’ tumor 1). All four factors were markedly induced but to different extents (Fig. 1B). While *Lhx1* was induced at the mesoderm stage and less so using the IM1 to IM5 conditions, *Pax2* was strongly induced by both IM1 and IM2 conditions compared to the others. *Osr1* was induced by IM1, IM2, and IM5. *Wt1*, which is expressed in IM but continues to be expressed in the kidneys, supporting cell precursors of the gonad, and later in Sertoli and granulosa cells, was mostly induced by IM3 and IM4. Because the IM markers show distinct expression profiles using the five differentiation conditions, we then determined the levels of early gonadal markers that are normally expressed at the bipotential stage of the gonads (E10.5 to E11.5). These include *Lhx9*, *Cbx2*, *Pod1*/*Tcf-21*, *Fog2*/*Zfpm2*, and *Gata4* (*28*). All these factors were strongly induced in IM3 and to lesser extent under IM4 conditions but at much lower levels under IM1, IM2, and IM5 conditions (Fig. 1B). Immunostaining of cells throughout the differentiation with antibodies against WT1 and GATA4 indicated high levels of these proteins at the IM3 stage, consistent with the qPCR experiments (Fig. 1C). We conclude that IM3 is the optimal condition to differentiate the mESCs toward early gonadal progenitors.

We also examined the expression levels of three additional TFs that are major markers of Sertoli cells and involved in Sertoli cell specification: *Sox9*, *Nr5a1*, and *Dmrt1*. All five IM conditions resulted in strong induction of *Sox9*, one of the most critical TFs of Sertoli cells, but one that is not unique to these (Fig. 1B). A strong expression of SOX9 was also evident at the protein level (Fig. 1C). *Nr5a1* and *Dmrt1* were not induced under any of the five IM conditions (Fig. 1B). To examine whether this differentiation protocol give rise to other gonadal cell types, notably to germ cells, Leydig cells, or peritubular myoid cells, we stained differentiated cells using markers of each of these lineages [DDX4 for germ cells, 3β Hydroxysteroid dehydrogenase (3β-HSD) for Leydig cells, and α–smooth muscle actin (αSMA) for peritubular myoid cells] and did not see expression of any of these markers (fig. S1).

### Transcriptomic analysis of the in vitro–derived early gonadal progenitors

To further verify the gene expression patterns throughout the differentiation protocol in a more comprehensive and unbiased manner, we performed RNA-seq analysis on the cells. Principal components analysis (PCA) showed the marked changes between ESC, EpiSC, M (mesoderm), and the five IM conditions (IM1 to IM5), which cluster closely together (fig. S2A). PCA of only IM1 to IM5 supported the results seen by the qPCR that IM3 and IM4 are similar to each other and distinct from the other treatments (fig. S2B). The 100 most differentially expressed (DE) genes (50 most up-regulated and 50 most down-regulated) between IM1/2/5 and IM3/4 conditions included several interesting markers (fig. S2C), such as the pluripotency marker *Sox2*, which is strongly expressed in ESC and EpiSC as expected. The three mesodermal markers *T*/*Bra* (*Tbxt*), *Cdx2* (*Caudal Type Homeobox 2*), and *Eomes* (*Eomesodermin*) were induced at the mesoderm stage. The IM/Sertoli marker *Wt1* was strongly expressed in IM3/4 and much less in IM1/2/5 (fig. S2C), consistent with the qPCR analysis (Fig. 1B). Last, the two metanephric mesenchyme markers *Six2* (*SIX homeobox 2*) and *Hoxd11* (*Homeobox D11*) were more highly induced in IM2/5 compared to IM3/4 (fig. S2C). We next compared expression of known markers of the following lineages: ESC, EpiSC, mesoderm, PM, lateral plate mesoderm (LPM), IM, genital ridge, adrenal, kidney, Sertoli cells, granulosa cells, and Leydig cells (gene list in data file S1) and correlated expression of these markers with the various differentiation conditions (fig. S2D). As expected, the pluripotency markers *Sox2*, *Nanog*, *Pou5f1*/*Oct4*, and *Klf4* were highly expressed at the ESC and EpiSC stages. The EpiSC-specific markers *Fgf5* and *Lin28b* were induced at the EpiSC stage. The mesodermal factors *T/Bra/Tbxt*, *Eomes*, *Tbx6*, *Cdx2*, and *Mesp1* were strongly induced at the M stage. Markers of the kidney and LPM lineages were induced under the IM1/IM2/IM5 conditions. In contrast, markers of the genital ridge (e.g., *Wt1*, *Lhx9*, *Tcf21/Pod1*, *Cbx2*, *Gata4*, *Emx2*, and *Zfpm2/Fog2*), and Sertoli cells (e.g., *Sox9*, *Fgf9*, *Erbb4*, *Cyp26b1*, *Cst9*, *Sox8*, and *Shbg*) were highly induced in IM3 with slightly lesser induction at IM4. Transcripts from *Sry* were not detected in any condition. In vivo, *Sry* shows a characteristic centre-to-pole wave of expression, and it is present very transiently in each cell (perhaps less than 3 or 4 hours) (*52*); this might be recapitulated under these in vitro conditions, making *Sry* more difficult to detect.

### Comparing in vitro–derived gonadal progenitors with in vivo gonadal cells

**Fig. 2.**
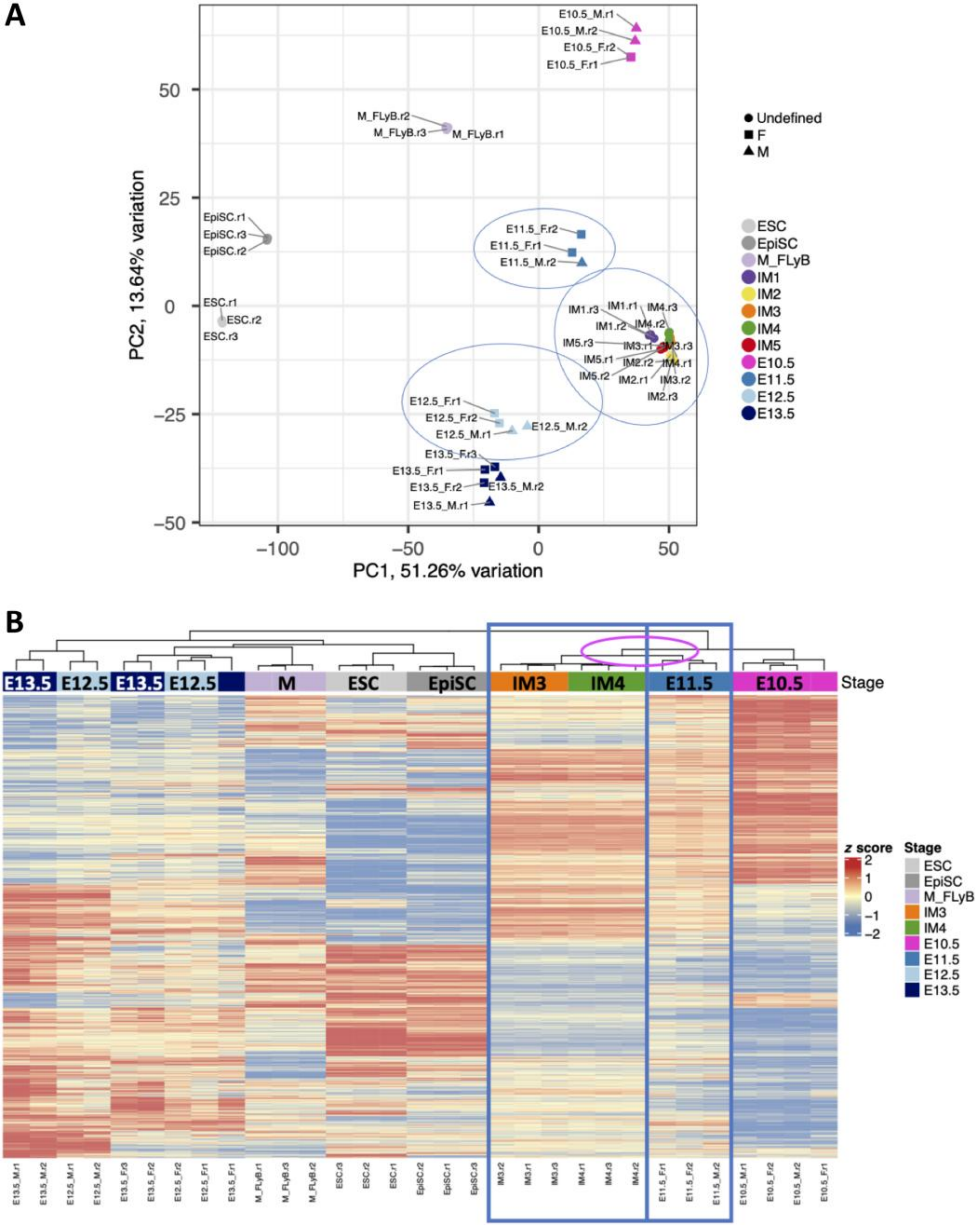
Comparing the transcriptome of in vitro–derived gonadal cells with bulk RNA-seq of in vivo gonadal cells. (**A**) PCA of selected samples based on normalized mRNA expression level after batch correction. The top 2 PCs are shown. E10.5 to E13.5 are gonadal cells isolated from entire embryonic male (triangle) or female (square) gonads (*16*). Three biological replicates were analyzed for each sample type. (**B**) Heatmap of the genes most DE between the E10.5 and E13.5 XY male gonads following batch correction (*16*). IM3/IM4 cluster closely to the E11.5 in vivo gonadal cells. Gene-level expression across samples is shown as a *z* score running from red (high) to blue (low). Three biological replicates were analyzed for each sample type.

The combination of qPCR, immunostaining, and RNA-seq analysis strongly supports the hypothesis that IM3 differentiated cells correspond to early gonadal progenitors. To verify that this is the case, we compared our bulk transcriptomic data with bulk RNA-seq of mouse XY and XX gonads at E10.5, E11.5, E12.5, and E13.5 (*16*). Comparing in vivo– versus in vitro–derived cells, the immediate and major change at principal component 1 (PC1) was the separation of the in vitro versus the in vivo datasets (fig. S3A). To address this, we either examined the PC2/3 data (fig. S3B) or used computational tools to correct the data as detailed in Materials and Methods (Fig. 2A). The PC1/2 of the corrected data (Fig. 2A) and the PC2/3 of the noncorrected data (fig. S3B) indicated that the IM condition cells cluster closely with early gonadal progenitors of E11.5. As the Zhao *et al.* dataset (*16*) of XY and XX at E12.5-E13.5 clusters relatively close together, we also performed PCA of the Zhao *et al.* data alone and this indicates the divergence between XY and XX cells from E12.5 onward (fig. S3C). On the basis of the Zhao *et al.* dataset (*16*), we chose the genes most DE between E10.5 and E13.5 XY gonads, as these are the genes induced during gonadal differentiation. Analyzing these genes in the different in vivo– and in vitro–derived cells demonstrated that E11.5 bipotential gonads cluster with IM3 and IM4 cells, indicating their similar gene expression patterns (Fig. 2B). The Poisson distance heatmap based on DE genes between E10.5 and E13.5 XY gonads shows a similar trend with IM3/4 being the most similar to early gonads (fig. S4A). Similar results were obtained both for Poisson distance heatmap (fig. S4B) and for differential gene expression analysis (fig. S4C) by selecting the most DE genes between E10.5 and E13.5 in XX gonads. Together, these data suggest that the IM3/IM4 conditions generate cells that are similar to E11.5 gonadal progenitors of the XY and XX gonads and can thus potentially serve as an optimal platform to generate all the somatic cells of the gonad.

### Inducing embryonic Sertoli cells from in vitro–derived gonadal progenitors

Although several Sertoli cell markers were induced by the IM conditions, flow cytometry analysis indicates that no CFP was apparent in these cells (fig. S5A), suggesting that Sertoli cell differentiation was incomplete. Hence, we next examined whether we can further differentiate the early gonadal progenitors toward the embryonic Sertoli cell lineage. Forced expression of *Sox9*, *Wt1*, *Gata4*, *Nr5a1*, and *Dmrt1* was shown to induce fibroblasts to differentiate into embryonic Sertoli-like cells (*40*). This, along with other studies, suggests that these five TFs are the critical players for Sertoli cell fate (*28*). Three of the five TFs (*Gata4*, *Wt1*, and *Sox9*) were induced in our differentiation protocol using growth factors, yet *Nr5a1* and *Dmrt1* were not (Fig. 1, B and C). Therefore, we examined whether forced overexpression of *Nr5a1* and *Dmrt1* can induce embryonic Sertoli cell differentiation from the early gonad progenitors. To that aim, we used doxycycline-inducible expression vectors and generated lentiviruses that overexpress either factor. IM3 cells were infected with either *Nr5a1*/*Dmrt1*-expressing lentiviruses or a combination of both and doxycycline was added for 4 days (fig. S5B). Overexpression of *Dmrt1* alone did not lead to expression of the TESCO-CFP reporter, while overexpression of *Nr5a1* alone resulted in weak CFP signals in several cells (fig. S5B). Overexpression of both *Dmrt1* and *Nr5a1* resulted in strong expression of the TESCO-CFP reporter (fig. S5B). Analyzing the cells using flow cytometry indicated that, compared to control IM3 cells (without overexpression of the two factors; fig. S5A, left), ~90% of IM3 cells with forced expression of *Nr5a1* and *Dmrt1* were CFP positive (fig. S5A, right). RNA-seq data showed that the viral induction was efficient, as these cells strongly overexpressed the *Nr5a1* and *Dmrt1* transcripts (fig. S5C).

Upon further passaging of the cells, they started forming tubule-like structures, reminiscent of testis cords. Many of the tubule-forming cells were CFP positive and are therefore likely to resemble Sertoli cells (Fig. 1A and fig. S5B, right).

To determine the similarity between the CFP-positive cells to embryonic Sertoli cells, we sorted them by flow cytometry and isolated RNA, which was analyzed by RNA-seq. Because the dataset of Zhao *et al.* (*16*) used entire XY and XX gonads, rather than purified Sertoli cells, we henceforth used the dataset from Maatouk *et al.* (*53*), which performed RNA-seq of E15.5 Sertoli cells, fluorescence-activated cell sorting (FACS)–sorted from the TESCO-CFP strain. We generated a list of DE genes between E13.5 XY and XX gonads as this should indicate Sertoli versus granulosa cell markers. These DE transcripts indicated that the induced Sertoli-like cells (iSLCs) cluster next to the E15.5 Sertoli cells, although the datasets are not overlapping (fig. S6A). A Poisson distance heatmap generated from the same transcripts indicates that the iSLCs are more similar to E12.5 and E13.5 male gonads (fig. S6B, black arrows) than to E12.5 and E13.5 female gonads (fig. S6B, white arrows).

### Analysis of the expression during the in vitro differentiation from ESCs to iSLCs

To better characterize the transition of mESC toward EpiSC, M, IM1 to IM5, and iSLCs, we performed a differential expression analysis and classified the genes according to their expression dynamics along the cell reprogramming (fig. S7, gene lists at data files S2 and S3). We performed gene ontology (GO) term enrichment analysis to identify the biological function associated with the genes overexpressed in the mESCs (P11), the M (P5), and the iSLC (P1-3-9-14), respectively (fig. S7 and data files S4 to S6). The genes overexpressed in ESC (P11) show enrichment of “Stem cell population maintenance” among the top GO term consistently with their identity. Among the top enriched GO terms of the mesodermal cells (P5) appear “mesoderm development” with enrichment of *Lhx1*, *Eomes*, and other mesodermal markers (data file S5). Last, GO term enrichment from the iSLCs showed “urogenital system development” as the most enriched term. Among the genes enriched for the urogenital system term, we find genes known to be expressed and involved in gonadal development as *Tcf21* (*Pod1*), *Notch 1/2*, *Gli2*, *Jag1*, *Gdnf*, *Smad3*, and *Fgf2*. These results indicate that the ectopic expression of *Nr5a1* and *Dmrt1* directed the differentiation of the IM cells toward gonadal cells (fig. S7 and data files S4 to S6).

### Comparing transcriptomes of in vitro–derived gonadal-like cells with cells isolated directly from in vivo gonads

Although the comparison to the bulk gonadal RNA-seq was informative, this dataset contains a mixture of gonadal cells including progenitor cells, germ cells, Sertoli cells, Leydig cells, peritubular myoid cells, and others (*16*). We therefore wished to compare our transcriptome data to fetal gonadal scRNA-seq data instead of bulk RNA-seq. The single-cell dataset of Stevant *et al.* (*15*) seems the most suited to be compared with bulk RNA-seq data as RNA were sequenced with a comparable methodology (full-length mRNA sequencing, ~15 million paired-end reads), unlike the data generated with the 10X Genomics technology (3′-end mRNA, shallow sequencing) (*54*, *55*). As described in the original paper, the cells are classified into six populations: early progenitors, interstitial progenitors, pre-Sertoli cells, Sertoli cells, Leydig cells, and endothelial cells (for extended data, see Materials and Methods and fig. S8, A to C).

**Fig. 3.**
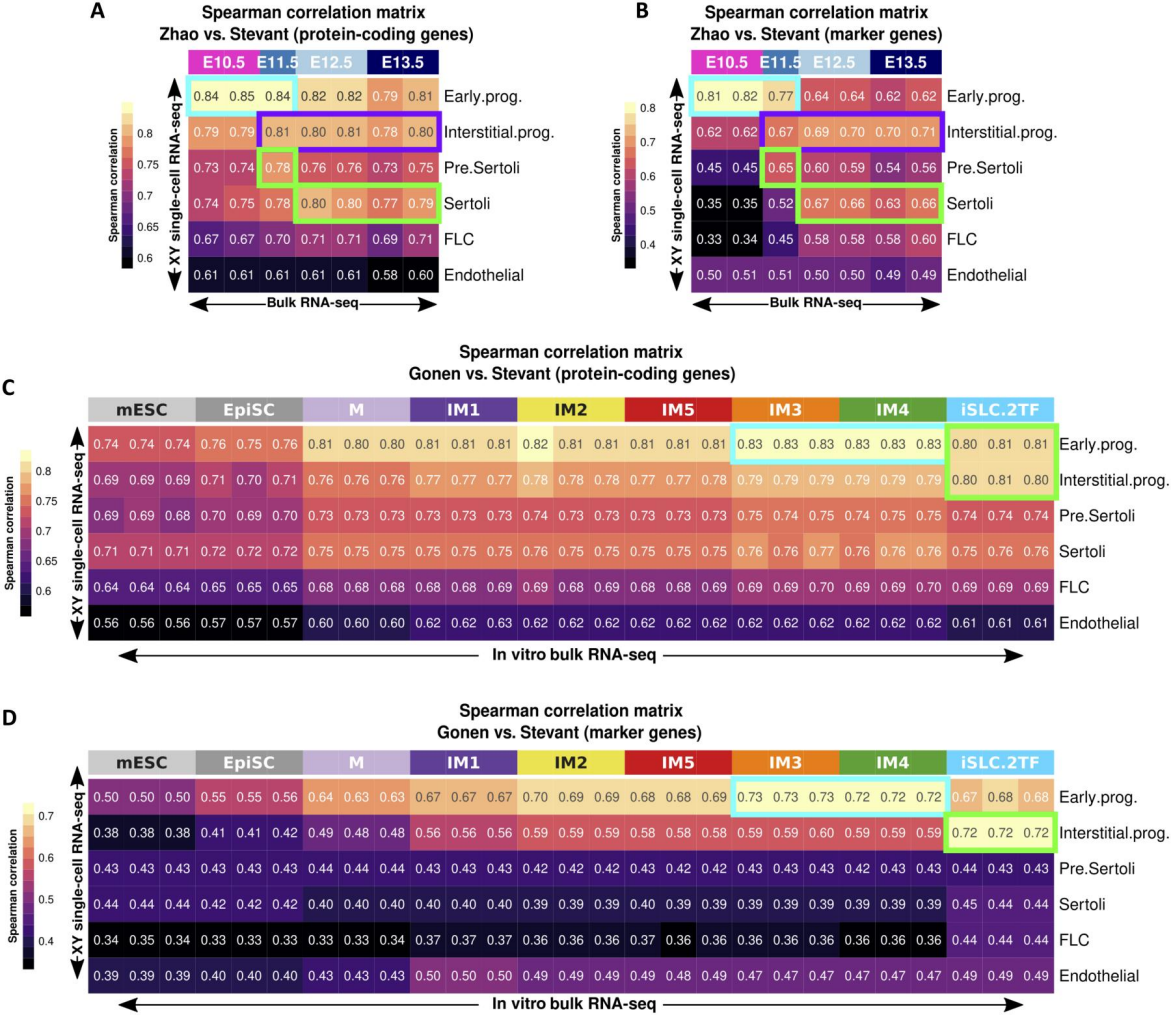
Comparing the transcriptome of in vitro–derived gonadal cells with scRNA-seq of in vivo gonadal cells. (**A** and **B**) Spearman correlation of the XY NR5A1^+^ gonadal scRNA-seq data transformed as pseudobulk per cell population (*15*) with the XY whole-gonad bulk RNA-seq (*16*) using the protein-coding genes (A) or a set of marker genes (B) (the most overexpressed genes from each of the six cell populations from the scRNA-seq data). The blue box points toward the high correlation of the early progenitor cells with E10.5/E11.5 bulk RNA-seq data, while the purple box indicates high correlation between interstitial progenitors and E11.5 to E13.5 bulk RNA-seq data. The green boxes show the correlation of the pre-Sertoli and Sertoli cells with E11.5 to E13.5 bulk RNA-seq data. FLC, Fetal Leydig cells. (**C** and **D**) Spearman correlation of the XY NR5A1^+^ gonadal scRNA-seq data transformed as pseudobulk per cell population (*15*) with the bulk RNA-seq data of the reprogramming mESCs using the protein-coding genes (C) or a set of marker genes (D). The green boxes point the correlation of iSLC with the early and/or interstitial progenitor cells, the blue boxes show the correlation of the IM3 and IM4 cells with the early progenitors.

To be able to compare bulk RNA-seq with the scRNA-seq data, we generated pseudobulks from the six identified cell clusters (see Materials and Methods), including the endothelial cells that will serve as negative control. We compared the gene expression of the bulk and the scRNA-seq data by performing pairwise Spearman correlations on all the protein-coding genes (Fig. 3, A and C) and on a set of marker genes (Fig. 3, B and D) of the six cell clusters (the most overexpressed genes from each cell cluster) (data file S7). We first compared the single-cell data of Stevant *et al.* (*15*) with the whole XY gonad RNA-seq of Zhao *et al.* (*16*) to see whether the scRNA-seq and the bulk dataset are comparable despite the differences in technology (Fig. 3, A and B). This revealed that the E10.5 and E11.5 whole gonads have a very high correlation with the scRNA-seq of early progenitors using both the protein-coding genes and the marker genes (blue boxes), which is consistent with the fact that the E10.5 and E11.5 gonads are mainly composed of these progenitor cells. Both comparisons (Fig. 3, A and B) show that the pre-Sertoli cells have a high correlation with E11.5 and that similarly, the Sertoli cells have high correlations with E12.5 and E13.5 whole gonads (green boxes), which correspond to their respective embryonic stages. We conclude that despite the technological differences and the heterogeneous cell composition of the bulk RNA-seq, the scRNA-seq and the bulk RNA-seq data are comparable.

We then performed the comparison between the RNA-seq performed along the mESC reprogramming and the scRNA-seq data using both the protein-coding genes and the list of marker genes (Fig. 3, C and D). We see that the IM3 and IM4 show a strong correlation with early progenitor cells in both comparison (Fig. 3, C and D, blue boxes). We can also observe that in both comparisons, the iSLCs show a high correlation with both the early and interstitial progenitors, reflecting the similarity of their transcriptomes (Fig. 3, C and D, green boxes). These two analyses confirmed that the IM and the iSLCs are acquiring a cell identity that is similar to gonadal progenitor cells.

Together, these data represent the first approach at generating early gonadal progenitors that have then been compared to cells from in vivo–derived gonads. The comparison to both bulk RNA-seq and scRNA-seq indicates that our in vitro–derived cells are highly similar to gonad progenitors and hence the in vitro–derived cells constitute excellent starting material for further differentiation of different somatic gonadal cell types. Our iSLCs activate the TESCO-CFP reporter, express Sertoli markers, and can form tubule-like structures, reminiscent of Sertoli cells, yet they are not fully identical to embryonic Sertoli cells.

### Deriving supporting cells of the gonad from hiPSCs derived from 46,XY male, 46,XX female, and a patient with 46,XY DSD

We next determined whether it was possible to derive human iSLCs (hiSLCs) from hiPSCs using these protocols to model a pathogenic variant, which causes male-to-female sex reversal. To differentiate the hiSLCs, we used the protocol optimized on mouse ES cells with minor modifications to the medium composition (detailed in Materials and Methods). We used three different hiPSC lines (two clones each): two from healthy individuals (46,XY male and 46,XX female) and one from a patient with 46,XY DSD that carries the pathogenic p.Arg313Cys variant in *NR5A1*. Heterozygous pathogenic variants in *NR5A1* are one of the most common causes of 46,XY DSD. The phenotypes associated with *NR5A1* are highly variable and range from a complete absence of testis determination (46,XY gonadal dysgenesis) and female external genitalia to testes in individuals with typical male external genitalia but who are infertile (*56*, *57*).

**Fig. 4.**
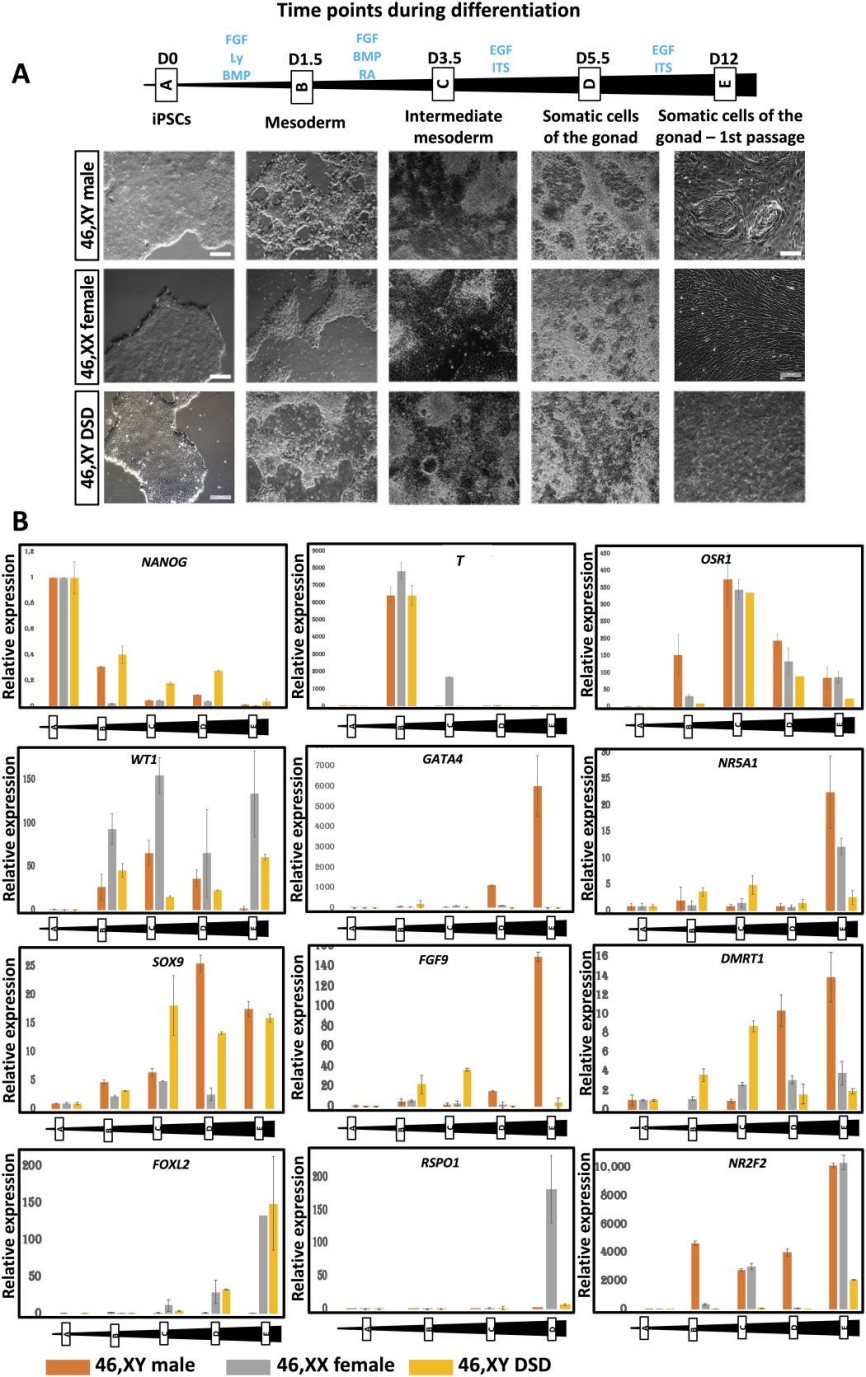
Differentiation of human iPSCs derived from healthy 46,XY male, 46,XX female, and a patient with 46,XY DSD with a pathogenic variant in *NR5A1*. (**A**) Differentiation time points are indicated together with BF images of the cells at each stage of the differentiation process. iPSC derived from the 46,XY healthy male are the only cells to self-aggregate and form tubule-like structures. D, days; scale bars, 200 μm. EGF, epidermal growth factor; ITS, Insulin-Transferrin-Selenium. (**B**) Representative RT-qPCR expression profiles of selected differentiation markers of the three iPSC-derived cell lines during the differentiation process.

During the differentiation process, cells exhibit changes in shape and transcript levels (Fig. 4, A and B). After 12 days of sequential culture in defined medium, cells from the control XY male show rearrangement and aggregation in the dish (Fig. 4A), which is not visible for the two other lines.

Real-time qPCR (RT-qPCR) of the three cell lines (Fig. 4B and fig. S9) reveals that expression of the pluripotency marker *NANOG* gradually reduces during the differentiation process. The cells differentiate into mesoderm and then IM as demonstrated by the expression of *T* (*TBXT*/*BRA*) and *OSR*1, respectively (Fig. 4B and fig. S9). *WT1* expression showed a gradual reduction in the 46,XY-derived cell cultures but persisted in those derived from 46,XX and 46,XY DSD cells (Fig. 4B and fig. S9). As differentiation continues, the somatic cells derived from 46,XY hiPSCs express markers specific to the somatic cells of the male gonad (*GATA4*, *NR5A1*, *DMRT1*, *SOX9*, and *FGF9*), whereas the 46,XX cells express markers specific for the somatic cells of the female gonad (*FOXL2* and *RSPO1*) (Fig. 4B and figs. S9 and S10). The somatic cells derived from a patient with 46,XY DSD carrying the *NR5A1* variant p.Arg313Cys show aberrant expression of gonadal transcripts with either a reduction (*NR5A1* and *DMRT1*) or an absence (*GATA4* and *FGF9*) of expression of Sertoli markers, and at the same time, there is increased expression of the granulosa marker *FOXL*2 (Fig. 4B and fig. S9). The cells derived from 46,XY DSD hiPSCs fail to express *FGF9* even though *SOX9* is expressed, suggesting a breakdown in establishment of the SOX9/FGF9 feed-forward loop required for the maintenance of Sertoli cell identity. The somatic cells derived from both 46,XX and 46,XY iPSC express *NR2F2*, a marker of presumptive steroidogenic cells, which along with steroidogenic enzymes *HSD3*β*1* and *STAR1* shows a statistically significant reduction in expression in the cells derived from 46,XY DSD hiPSCs (Fig. 4B and fig. S9).

### Functional and structural characterization of the somatic cells derived from hiPSCs

**Fig. 5.**
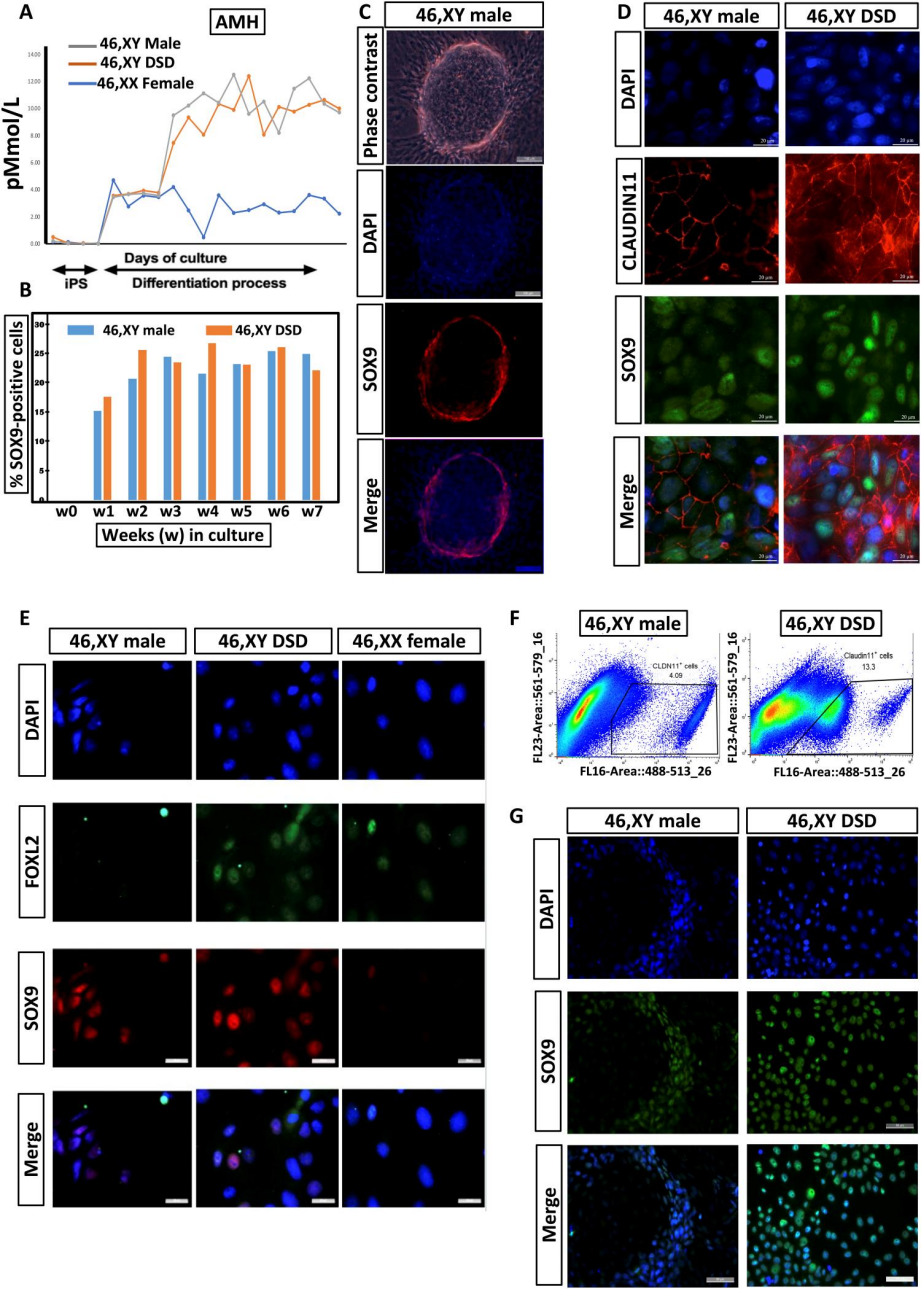
Characterization of iSLCs from 46,XY male and patient with 46,XY DSD. (**A**) The Sertoli cell product, AMH, is secreted into the medium at comparable levels by the iPSC-derived from 46,XY male and 46,XY DSD cells during differentiation and at lower levels by 46,XX cells. (**B**) Continuous and prolonged expression of SOX9 in the differentiating 46,XY male and 46,XY DSD cells. (**C**) Top: Phase contrast of iPSC-derived from 46,XY cells after 12 days of sequential culture in conditioned media shows spontaneous formation of tubular structures. Middle and bottom: The tubule-like structures are composed of SOX9-positive cells; scale bars, 100 μm. (**D**) Expression of SOX9 and CLAUDIN-11 in iPSC-derived cells from 46,XY healthy male and patient with 46,XY DSD after 12 days of sequential culture in defined media. Both cell lines express SOX9 and CLAUDIN-11, but the spatial organization of CLAUDIN-11 is disrupted in iPSC-derived cells from the patient with 46,XY DSD; scale bars, 20 μm. (**E**) Immunocytochemistry for SOX9 and FOXL2 in iPSC-derived cells from 46,XY healthy male, 46,XX female, and patient with 46,XY DSD after 12 (+5) days of sequential culture in defined media. The iPSC-derived 46,XY male cells express SOX9 but not FOXL2, whereas the iPSC-derived 46,XX cells express FOXL2 but not SOX9. In contrast, a proportion of the iPSC-derived 46,XY DSD cells express both SOX9 and FOXL2 within the same cell; scale bars, 20 μm. (**F**) Flow cytometric–sorted iPSC-derived CLAUDIN-11-positive cells from a 46,XY male and a patient with 46,XY DSD following 12 (+12) days of sequential culture in defined media. (**G**) After FACS, the iPSC-derived CLAUDIN-11-positive cells from a 46,XY male and a patient with 46,XY DSD are seeded onto Matrigel in a chamber slide. After 7 to 10 days, the 46,XY male cells spontaneously organize into circular tubular structures, whereas 46,XY DSD cells do not form these structures. Both cell lines express SOX9; scale bars, 50 μm.

AMH, which is required for the regression of the Müllerian duct derivatives (*58*), is secreted by fetal Sertoli but not early granulosa cells. Consistent with this, somatic cells derived from 46,XY iPSCs secrete AMH (Fig. 5A). The 46,XY DSD cells also secrete AMH. This is expected because the patient with DSD has rudimentary Müllerian structures (*56*), indicating limited Sertoli cell function, where enough AMH was secreted to cause partial Müllerian derivative regression but not enough for testis formation resulting in testicular dysgenesis. The 46,XX cells secrete low levels of AMH (Fig. 5A). Depending on the follicular stage, varying levels of AMH are secreted by granulosa cells in developing human ovaries. On prolonged culture (for over 7 weeks), cells derived from both 46,XY and 46,XY DSD hiPSCs continued to express the SOX9 protein (Fig. 5B) and other Sertoli cell genes (WT1 and DMRT1; fig. S10). Following 12 to 15 days of sequential culture in defined media, the cells derived from 46,XY hiPSCs spontaneously form tubular structures (Fig. 5C, top, and fig. S11). These tubular structures are enclosed by SOX9-positive cells (Fig. 5C, lower panels, and fig. S11), further suggesting that they are Sertoli-like cells. In the developing testis, CLAUDIN-11 is a critical transmembrane component of Sertoli cell tight junctions and is imperative for initiation and maintenance of functional Sertoli cell differentiation (*59*). Murine Sertoli cells lacking *Claudin-11* can proliferate and maintain the expression of testis cell markers; however, they acquire a fibroblast phenotype, lose tight junction integrity, and are eliminated through the lumen (*60*). The Sertoli-like cells derived from 46,XY and 46,XY DSD hiPSCs express both SOX9 and CLAUDIN-11 (Fig. 5D). However, unlike the cells derived from 46,XY hiPSCs, those derived from 46,XY DSD hiPSCs do not show the organized spatial distribution of CLAUDIN-11 surrounding the SOX9-expressing cells. This leads to the disruption of tight junctions resulting in absence of tubule formation (see below). The formation of organized tubular structure by the cells derived from 46,XY hiPSCs and their absence in those derived from 46,XY DSD hiPSCs was also confirmed by 3D confocal imaging of coexpression of VIMENTIN and SOX9 (fig. S12 and movies S5 and S6). The somatic cells derived from 46,XY hiPSCs express SOX9, and those from the 46,XX hiPSCs express FOXL2. In contrast to the cells derived from control individuals, those from the 46,XY DSD iPSCs express both the pro-testis SOX9 and pro-ovary FOXL2 proteins (Fig. 5E). Similar cell populations were identified in adult murine testes after *Dmrt1* ablation (*61*). These cells possibly represent multipotent progenitors or cells transitioning from Sertoli cells to granulosa cells in the trans-differentiating gonad.

During the differentiation process from 46,XY and 46,XY DSD hiPSCs, CLAUDIN-11 is expressed in SOX9-positive cells (Fig. 5D). We sorted CLAUDIN-11–positive cells by flow cytometry following 12 (+12) days of sequential culture in supplement-enriched media (Fig. 5F). The sorted cells express SOX9 and were seeded on Matrigel-coated chamber slides. After 7 to 10 days in culture, the 46,XY cells spontaneously organize into circular tubular structures, whereas 46,XY DSD cells are unable to form organized structures (Fig. 5G), possibly because of disruption in organization of CLAUDIN-11.

**Fig. 6.**
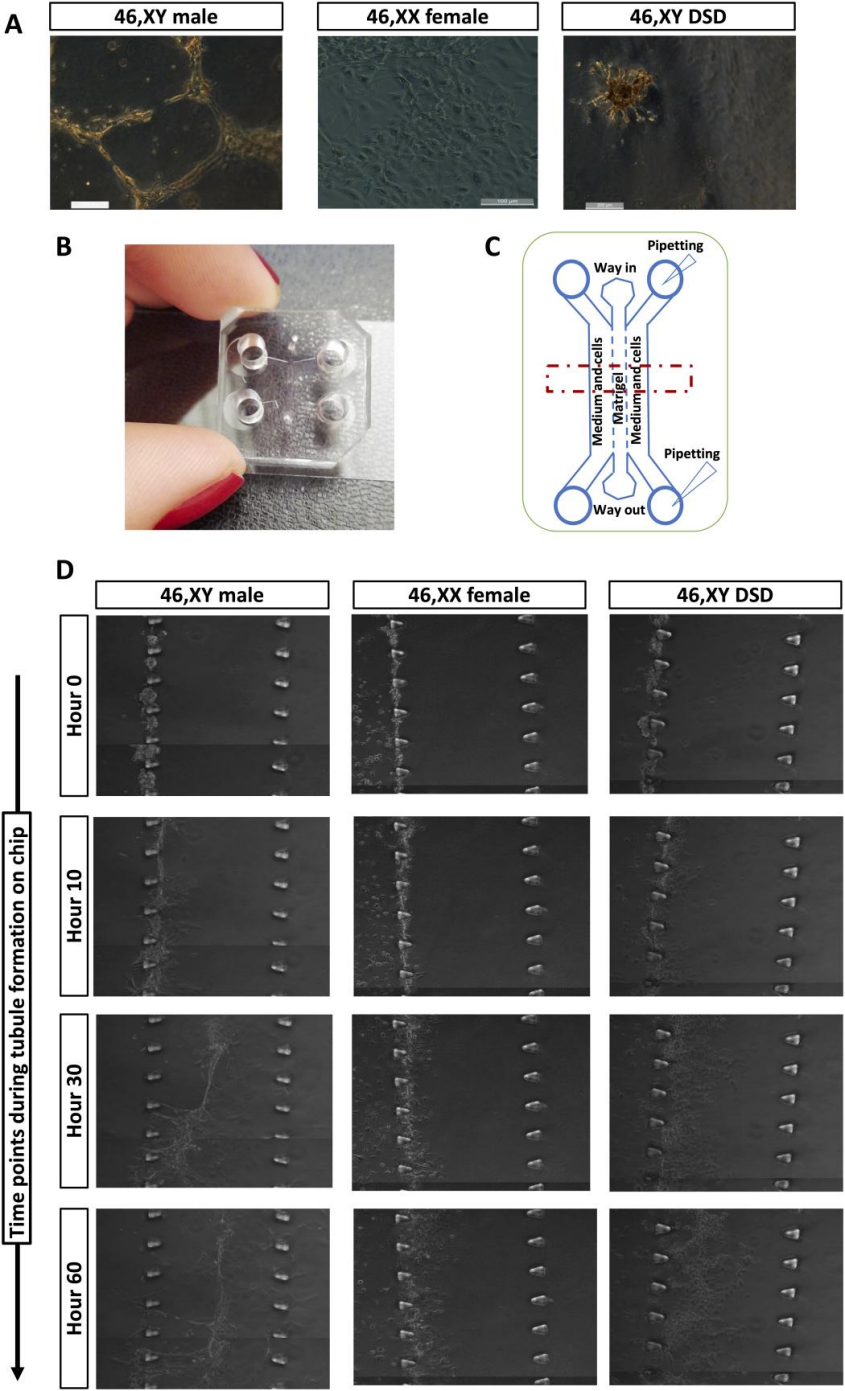
3D tubule formation in a microfluidic chip. (**A**) After FACS, the iPSC-derived CLAUDIN-11-positive cells from 46,XY male, 46,XX female, and 46,XY DSD cells were expanded for 7 to 14 days in Sertoli cell medium. These cells were then seeded on a constrained space on hardened (50%) Matrigel domes. The iPSC-derived cells from the 46 XY male migrate and form tubular structures, whereas 46,XX female and 46,XY DSD cells do not form tubular structures; scale bars, 100 μm for the panels representing cells from 46,XY male and 46,XY DSD. Scale bars, 200 μm for the panels representing cells from 46,XX female. (**B**) PDMS microfluidic chip designed for 3D tubule formation (GONAChip) together with (**C**) a schematic representation of the chip. (**D**) Still images from time-lapse video of cell migration, aggregation, and tubule formation on GONAChip. The time-lapse videos (movies S1 to S3) show that the cells from all the three iPSC-derived cell lines can proliferate. The iPSC-derived 46,XX cells do not migrate, whereas 46,XY male and 46,XY DSD cells migrate and enter the central chamber lined with Matrigel (hours 0 to 30). The iPSC-derived 46,XY male cells organize and form distinct tubular structures, whereas the iPSC-derived 46,XY DSD cells do not form tubular structures (hours 30 to 72).

To further assess the ability of hiPSC-derived supporting cells to form tubular structures, we seeded the sorted cells (after expansion for 2 to 3 weeks) atop a soft substrate made of Matrigel (50%, v/v). The cells derived from 46,XY iPSCs can form tubular structures, whereas those derived from 46,XX and 46,XY-DSD hiPSCs aggregate but fail to form tubular structures (Fig. 6A and fig. S13). To evaluate the capacity of supporting cells derived from iPSCs to migrate, aggregate, and form 3D structures, we designed a polydimethylsiloxane (PDMS) microfluidic device termed GONAChip (Fig. 6B) with three channels: one central for the Matrigel loading and two lateral channels for media and cell loading, respectively (Fig. 6C). Gaps in the channel walls provide a medium/Matrigel interface on which cells where cultured. After sorting, the CLAUDIN-11–positive cells from 46,XY, 46,XX, and 46,XY DSD lines were expanded for 2 to 3 weeks. Cells were then seeded on one lateral channel of GONAChip. The seeded devices where then incubated in a humid chamber at 37°C in 5% CO2 for 72 hours. Live imaging (movies S1 to S3) shows that cells from all the three cell lines can survive and proliferate. We found that 46,XY and 46,XY DSD cells migrate and invade the central channel filled with Matrigel (hours 0 to 30), whereas the 46,XX cells do not. We also found that 46,XY cells seem to migrate in a coordinated manner that leads to recognizable tubular structures. On the other hand, 46,XY DSD invasion of Matrigel appears less coordinated (hours 30 to 60) and the cells do not form tubular structures (Fig. 6D).

We assessed the cell velocity using the scratch-wound assay. Differentiated cells derived from iPSCs from 46,XY male and 46,XY DSD were plated at a density of 50 × 10^5^ cells per well in a 96-well ImageLock microplate, and a thin wound was created, 24 hours after seeding, by scratching the culture with WoundMaker Tool. The wound closure was measured by calculating the decrease in the wound width over time (table S4 and fig. S14A). Statistical analysis of the slope generated under the different conditions shows similar organized movement for cells derived from a 46,XY healthy male that contrasts with the reduced movement of 46,XY DSD cells (fig. S14, B and C).

### CRISPR-Cas9 genome–edited 46,XY DSD cells resemble 46,XY cells

**Fig. 7.**
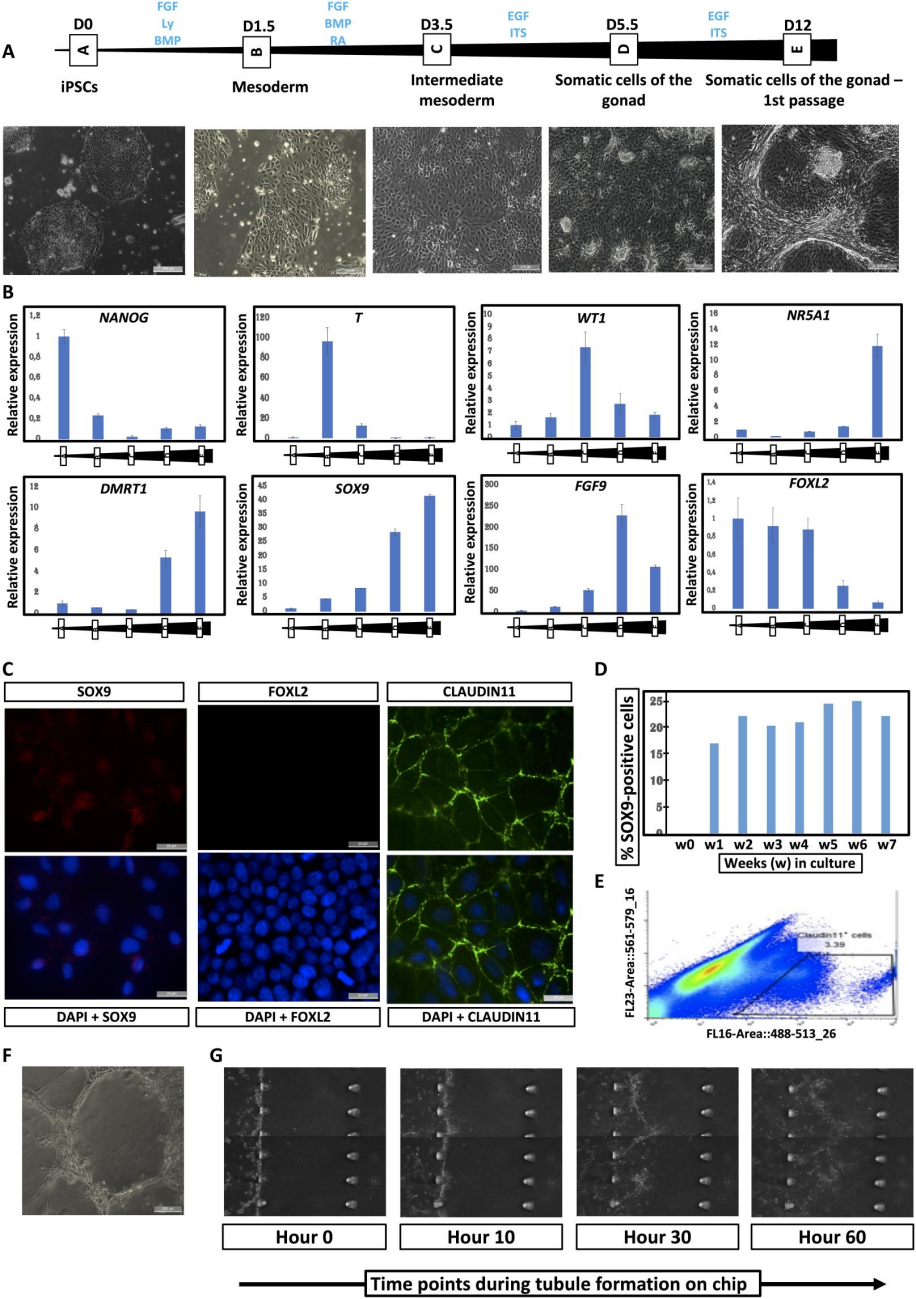
CRISPR-CAS9 correction of the *NR5A1* pathogenic variant in iPSC-derived 46,XY DSD cells restores iSLC properties. (**A**) Differentiation time points and BF images of the corrected 46,XY DSD cells during the differentiation process; scale bars, 200 μm. (**B**) RT-qPCR expression data of selected markers during the differentiation process. (**C**) Expression of SOX9, FOXL2, and CLAUDIN-11 in *NR5A1*-corrected 46,XY DSD iPSC–derived cells after 12 days of sequential culture in defined media. Cells express SOX9 and CLAUDIN-11, but the expression of FOXL2 is not detected; scale bars, 20 μm. Moreover, CLAUDIN-11 expression is spatially organized, similar to that seen in 46 XY, male cells (Fig. 5D). (**D**) Continuous and prolonged expression of SOX9 is observed in the differentiating cells *NR5A1*-corrected 46,XY DSD iPSC–derived cells. (**E**) Flow cytometric sorting of CLAUDIN-11-positive *NR5A1*-corrected 46,XY DSD iPSC–derived cells following 12 (+12) days of sequential culture in conditioned media. (**F**) After FACS, the CLAUDIN-11-positive *NR5A1*-corrected 46,XY DSD iPSC–derived cells were expanded for 7 to 14 days in Sertoli cell medium and then seeded on a constrained space on hardened (50%) Matrigel domes. The cells migrate and form tubular structures; scale bars, 100 μm. (**G**) Still images from a time-lapse video of cell migration, aggregation, and tubule formation on GONAChip (movie S4) show that the cells proliferate, migrate (hours 0 to 30), and self-organize to form distinct 3D tubular structures (hours 30 to 60).

We corrected the pathogenic variant in 46,XY DSD hiPSCs using CRISPR-Cas9 genome editing. The edited cells were then subjected to differentiation following the same protocols as for the 46,XY and 46,XY DSD iPSCs (Fig. 7A). The pluripotent cells (expressing *NANOG*) differentiate into mesoderm (expressing *T/BRA*) and IM (expressing *WT1*). As differentiation occurs, these cells express markers specific to the somatic cells of the male gonad (*NR5A1*, *DMRT1*, and *SOX9*) (Fig. 7, B and C, and figs. S9 and S10). The 46,XY DSD cells after correction of the *NR5A1* variant regain the expression of *FGF9*, while *FOXL2* expression cannot be detected (Fig. 7, B and C, and figs. S9 and S10). Moreover, the cells show spatial reorganization of CLAUDIN-11 and VIMENTIN similar to that of 46,XY cells (Fig. 7C, fig. S12, and movie S7). On prolonged culture (for over 7 weeks) the cells derived from this rescued cell line continue to express the SOX9 protein (Fig. 7D), WT, and DMRT1 (fig. S10).

The rescued cells were sorted using CLAUDIN-11 (Fig. 7E), expanded, and seeded atop Matrigel substrates as described above. These cells show spontaneous tubule formation similar to those derived from 46,XY hiPSCs (Fig. 7F and fig. S13). Using the GONAChip device, sorted CLAUDIN-11–positive rescued cells proliferated, migrated (hours 0 to 30), and self-organized to form distinct 3D tubular structures (hours 30 to 60) similar to those derived from 46,XY DSD iPSCs (images extracted from time-lapse videos; movie S4). Assessment of the cell velocity using the scratch-wound assay showed restoration of wound closure speed and organized movement similar to that of cells derived from a 46,XY healthy male (table S4 and fig. S14, B and C).

## DISCUSSION

Several attempts have been made to generate embryonic gonadal somatic cells in vitro either by forced expression of exogenous TFs (*40*) or by using combinations of growth factors and defined media (*43*–*45*). In most of these studies, the resulting populations were defined as Sertoli cells on the basis of expression of certain Sertoli-specific markers. However, they show a limited expression of endogenous Sertoli cell markers, with little to no expression of the key testis factor *Sox9*. Another differentiation strategy is the use of supplement-enriched media to differentiate human pluripotent cells toward the IM and associated coelomic epithelium (*62*) and further toward granulosa-like (*42*) or Sertoli-like cells (*46*, *47*). However, the in vitro–derived Sertoli-like cells also lack robust and prolonged expression of key Sertoli-cell markers (SRY, SOX9, NR5A1, and DMRT1) and their functionality was not explored. This precluded their use as a sufficiently accurate model to study naturally occurring variants causing errors in human testis determination.

Here, we have derived gonadal progenitors from mouse ESCs and human iPSCs following the in vivo developmental pathway for gonad formation. For differentiation of mESCs to IM, we optimized the culture conditions in which the medium contained bFGF, BMP, and RA but not the WNT agonist CHIR99021 or Activin A. The IM cells were further differentiated toward the early somatic progenitors of the gonad. In contrast to other studies, the transcriptomic profile of our in vitro–derived mouse gonadal cells strongly resembled that of E10.5 to E11.5 gonadal progenitors based on both bulk RNA-seq and scRNA-seq comparisons (*15*, *16*). The expression of three key TFs (*Gata4*, *Wt1*, and *Sox9*) was endogenously induced in the progenitor cells, whereas that of *Nr5a1* and *Dmrt1* was not. Forced overexpression of *Nr5a1* and *Dmrt1* in the in vitro–derived progenitors resulted in strong activation of the Sertoli cell–specific TESCO-CFP reporter (*49*) and formation of tubule-like structures resembling testis cords. The transcriptome of the CFP-positive in vitro–derived progenitor cells clustered with the in vivo TESCO-CFP E15.5 Sertoli cells; however, these were not identical. This could be because our in vitro–derived Sertoli-like cells are less mature than E15.5 Sertoli cells and comparisons with earlier stages, such as E12.5 or E13.5, may show higher degrees of similarity. Alternatively, it is possible that more factors are needed to direct the progenitor cells closer to a “Sertoli cell identity.” This opens an avenue for future research to explore conditions that promote derivation and maturation of Sertoli cells. In the future, it will be interesting to compare in vitro–derived and in vivo gonadal cells using scRNA-seq.

Using the culture conditions optimized with mESCs, we directed both 46,XY and 46,XX hiPSCs toward gonadal progenitors. Although similar growth factors were used, we observed differences in gene expression not only between the mouse- and human-derived cells but also between our hiPSCs-derived cells and other published studies. Notably, *Sry* transcripts were absent in all conditions tested using the murine cells although both *SRY* mRNA and protein were detected at low levels in XY hiPSCs and in the human XY in vitro–derived gonadal cells (fig. S15), respectively. This also contrasts with the study by Knarston *et al.* (*46*) where *SRY* mRNA expression was not detected in hiPSCs-derived Sertoli-like cells. SOX9 overexpression can drive testis differentiation in the absence of *Sry* in transgenic XX embryos (*63*) or when the *SOX9* gene or *SOX9*-specific regulatory elements are duplicated (*64*–*66*). It is possible that the supplement-enriched medium used by us for differentiation of mESCs and by others for differentiation of hiPSCs bypasses *SRY* expression and directly or indirectly up-regulates *SOX9*. However, we observed that cells derived from 46,XX hiPSC lines did not express Sertoli cell markers. This suggests that derivation of Sertoli-like cells from hiPSCs in our culture system is dependent on SRY as would usually occur in vivo. We also observed differences in the expression of two other key testis-determining genes *NR5A1* and *DMRT1*. These genes were not induced in the gonadal progenitors derived from mESCs. Similarly, in the study by Knarston *et al.* (*46*), *NR5A1* and *DMRT1* expression was either not induced or expressed at very low levels. This could be because induction of *DMRT1* expression is contingent on a threshold of SRY expression. In contrast to previous studies, we observe a robust and prolonged (for more than 7 weeks) expression of the key testis-determining genes *SOX9*, *NR5A1*, and *DMRT1* in hiPSC-derived Sertoli-like cells. In monolayer cultures, these cells secrete AMH, self-aggregate, and spontaneously form tubule-like structures reminiscent of testis cords. The tubular structures are lined with SOX9-positive cells surrounded by the tight junction protein CLAUDIN-11. Using a specially designed PDMS microfluidic chip, we demonstrated that CLAUDIN-11-sorted in vitro–derived Sertoli-like cells can migrate and self-aggregate to form 3D tubular structures.

Using the methods described here, it is possible to direct pluripotent cells from both mouse and human, to mimic the in vivo developmental pathway, and generate early gonadal progenitors and Sertoli-like cells using sequential defined media. This approach circumvents the need for continuous expression of exogenous factors involved in sex determination (*40*, *67*, *68*), which can override the endogenous signals and force these cells to develop into the somatic cells of the gonad. Allowing the cells to differentiate, based on their natural genetic composition, enables the investigation of naturally occurring gene variants of DSD that disrupt this process (*35*).

As a proof of principle, we differentiated iPSCs from a patient with DSD carrying a naturally occurring sex-reversing missense variant. The somatic cells derived from the patient’s iPSCs did not show appropriate stage-specific expression of Sertoli cell markers and lacked the ability to self-aggregate or form testis tubule-like structures. Correction of this missense variant by CRISPR-Cas9 in patient’s iPSCs was sufficient to reestablish the ability of the derived cells to form Sertoli-like cells that can aggregate to form tubular structures similar to the cells derived from 46,XY male.

This in vitro system can therefore be used to study human testis determination and the effects of naturally occurring variants that disrupt these processes and cause DSD. This is important as >50% of all DSD cases do not have an established genetic etiology and the phenotypic variability associated with variants in known DSD genes is currently unexplained (*35*). This would enable us to determine both the causality of novel genes proposed to cause DSD and explore the phenotypic variability observed between individuals with identical pathogenic gene variants.

Generation of early gonadal progenitors and Sertoli-like cells could be further refined to produce other somatic lineages of the gonad, such as Leydig and peritubular myoid cells of the testis or granulosa and theca cells of the ovary, as well as other gonadal lineages that are being identified by single-cell sequencing approaches (*14*, *15*, *54*). Recently, fetal ovarian somatic cell–like cells were derived from mESC, using an approach similar to ours. When these cells were cocultured with PGCLCs, reconstituted oocytes were formed that could undergo meiosis and produced live and healthy pups (*48*). The approaches that we have developed could be exploited to examine the capability of these cells to support in vitro spermatogenesis and oogenesis, leading to the understanding of a range of fertility-related issues (*50*). Our model is a powerful approach to study testis determination and differentiation in mouse and human as well as cellular and molecular effects of genes/variants associated with DSDs.

## MATERIALS AND METHODS

### Mice

All animals were maintained with appropriate care according to the United Kingdom Animal Scientific Procedures Act 1986 and the ethics guidelines of the Francis Crick Institute.

### ESC and iPSCs

We used mouse ESCs and hiPSCs to derive somatic cells of the developing gonad, using defined supplement-enriched medium. The details of cells lines and extended methodology are in the Supplementary Materials.

### Derivation and differentiation of mouse ESC from blastocysts

XY Mouse ESC (TESCO-CFP;R26-rtTA) were differentiated into epiblast-like cells as previously described (*50*). Early mesoderm differentiation was induced by a chemically defined medium supplemented with Polyvinyl Alcohol (CDM-PVA) (*51*, *69*) followed by IM induction on culture for 3.5 days in CDM-PVA media supplemented with combination of bFGF, BMP4, RA, CHIR99021, and Activin A (for IM1 to IM5). The cells were then cultured with viral particles containing Nr5a1 and Dmrt1 for 24 hours followed by “Sertoli media” (Ad-DMEM/F12 supplemented with P/S, L-Glu, Hepes, X1 B27 and N-Acetyl L-Lysine (nAC), mouse Epidermal Growth Factor (mEGF), recombinant human Follicle Stimulating Hormone (rhFSH), recombinant mouse Fibroblast Grwoth Factor 9 (rmFGF9), prostaglandin, testosterone, and Activin A). Isolation and validation of resultant populations were performed using FACS, quantitative RT-qPCR, and immunofluorescence.

### RNA sequencing

Total RNA extracted from triplicate of RNA samples of ESC, EpiSC-like cells, M-like cells, IM-like cells (IM1 to IM5), and CFP-sorted Sertoli-like cells using TRIzol. Libraries were prepared using the TrueSeq Library Prep kit V2 (Illumina) according to the manufacturer’s instructions. Sequencing was performed on the Illumina HiSeq 4000 system (paired end, 75 bp).

### Data collection

RNA-seq data from mouse embryonic gonad tissue (E10.5, E11.5, E12.5, and E13.5) and TESCO-CFP E15.5 Sertoli cells were downloaded from the National Center for Biotechnology Information’s Sequence Read Archive accession: SRP076584 (*16*) and SRP033562 (*53*), respectively. scRNA-seq data on embryonic mouse gonads were downloaded from the Gene Expression Omnibus (GEO) under accession GSE97519 (*15*).

### Alignments and abundance estimation

Cutadapt v1.9.1 was used to trim adapter sequences. Reads were aligned against the GRCm38 genome assembly with Ensembl release 86 transcript annotations using STAR v2.5.2a (*70*). Gene-level abundance estimates were calculated with RSEM v1.3.0 (*71*). A copy of the code used for this step (*72*) is freely available as a Nextflow pipeline and is available at Zenodo (https://zenodo.org/record/4270402#.Y3zrhOzP3a0) and the Github repository (https://github.com/crickbabs/BABS-RNASeq.git).

### Data exploration and differential expression

Differential expression analysis was performed in R 3.6.0 using Bioconductor package DESeq2 (*73*). Data were normalized for differing library size using DESeq2’s default method. Differential gene expression analysis between replicate groups was assessed using the default Wald test. Genes were called significant if they passed a combined filter of (i) false discovery rate ≤ 0.01, (ii) fold change ≥ ±2, (iii) base mean ≥ 100 from the Wald test results, and (iv) a mean normalized read count of ≥100 in at least one of the tested replicate groups.

PCA was used to assess the relationship between gene expression across samples using the PCA tools R package’s “pca” function. Data were variance-stabilized using DESeq2’s “vst” function before the PCA, and batch effect between in vitro*/*in vivoprotocols was corrected using the limma package. Sample similarity was assessed using a Poisson dissimilarity matrix constructed from the uncorrected normalized counts of all samples using the “PoissonDistance” function from the PoiClaClu package. Heatmaps were generated from variance-stabilized data and clustered using a “complete” clustering method on a set of Euclidean distances. Data were additionally scaled per gene using a *z* score to aid visualization.

### Exploration of the genes driving the change using differential expression analysis

Differential expression analysis between the in vitro–reprogrammed cells were performed using DEseq2 with the “LRT” test on library size normalized read counts. Genes presenting an adjusted *P* value < 0.05 were plotted as a heatmap using the Pretty Heatmap R package and clustered into 15 profiles using “ward.D2” algorithm (P1 to P15, data files S2 and S3). Genes with roughly similar expression profiles were merged and subjected to a GO term enrichment analysis using ClusterProfiler (Biological Processes). GO term similarity were reduced using the similarity function from ClusterProfiler. to remove redundant information (data files S4 to S6).

### Comparison with scRNA-seq data

ScRNA-seq data from Stevant *et al.* (*15*) (GSE97519) were mapped on the mouse reference genome (GRCm38) using GemTools, duplicated reads and nonuniquely mapped reads were discarded with SAMtools, and gene expression was assessed using an in-house pipeline as described by Stevant *et al.* (*15*).

Pseudobulks per cell types were generated by summing the read count per genes for each of the six cell clusters identified in the original study (*15*). For each comparison, pseudobulk and RNA-seq read counts were normalized using DESeq2 size factor. Comparison was performed using pairwise Spearman correlations and visualized as a heatmap using the Pretty Heatmap R package (default clustering parameters) using both the protein-coding genes and a set of marker genes for each of the cell types contained in the scRNA-seq data.

### hiPSC lines

The patient with 46,XY DSD met the revised criteria of the Pediatric Endocrine Society/European Society for Pediatric Endocrinology. We obtained written informed consent from the patient and family members who participated in the study. This study was approved by the local French ethical committee (2014/18NICB; registration no. IRB00003835). hiPSC lines were derived from peripheral blood mononuclear cells of a 46,XY girl carrying a de novo heterozygous p.Arg313Cys pathogenic variant in *NR5A1* (*56*), a healthy 46,XY male, and a healthy 46,XX female. A fourth line of iPSC was generated after correction of the *NR5A1* mutation, using CRISPR-CAS9, in the iPSCs derived from the 46,XY girl.

Twenty-four hours before start of differentiation, colonies of hiPSCs cultivated in feeder-free mTeSRPlus medium were dissociated into single-cell solution and plated at a high density. Similar to murine pluripotent cells, hiPSCs were subjected to serial differentiation in conditioned medium with minor modifications of the medium composition. Isolation and validation of resultant populations were performed using FACS, qRT-PCR, and immunocytochemistry. AMH was measured by a one-step sandwich enzyme-linked immunosorbent assay (Access AMH, Beckman Coulter Company) (*74*).

Formation of spontaneous tubular structures, by supporting cells derived from hiPSCs, was observed by plating the cells on top of domes made of hardened (50%) organoid-grade Matrigel cast onto a well of chilled 12-well plates. Resultant structures were captured with a Leica Microsystems DMI4000B microscope at ×40, ×63, and ×100 (with oil) magnifications.

To evaluate the capacity of hiPSC-derived supporting cells to migrate, aggregate, and form 3D structures, a three-channel PDMS microfluidic device, termed GONAChip, was designed. Confluent sorted cells were added to the inlets and allowed to migrate into the central channel lined with organoid-grade Matrigel. Time-lapse imaging was performed with pictures taken every 15 min for 70 hours and processed using the ZENlite Software (ZEISS). Cell velocity during migration was assessed using the scratch-wound assay according to the manufacturer’s protocol (Incucyte, Sartorius, Germany).

## Notes

View/request a protocol for this paper from *Bio-protocol*.
