# Supplementary material for: In vitro cellular reprogramming to model gonad development and its disorders
Source: Sci Adv. 2023 Jan 4;9(1):eabn9793. doi: 10.1126/sciadv.abn9793 (PMC9812383; doi:10.1126/sciadv.abn9793)
Supplement: Supplementary file 1 — Materials and Methods Figs. S1 to S15 Tables S1 to S3 [file sciadv.abn9793_sm.pdf]

Science Advances
AAAS

Supplementary Materials for

In vitro cellular reprogramming to model gonad development and its disorders

Nitzan Gonen *et al.*

Corresponding author: Nitzan Gonen, nitzan.gonen@biu.ac.il;
Kenneth McElreavey, kenneth.mcelreavey@pasteur.fr;
Robin Lovell-Badge, robin.lovell-badge@crick.ac.uk; Anu Bashamboo, anu.bashamboo@pasteur.fr

Sci. Adv. **9**, eabn9793 (2023)
DOI: 10.1126/sciadv.abn9793

The PDF file includes:

Materials and Methods
Figs. S1 to S15
Tables S1 to S3
Legend for table S4
Legends for data files S1 to S7
Legends for movies S1 to S7

Other Supplementary Material for this manuscript includes the following:

Table S4
Data files S1 to S7
Movies S1 to S7

Materials and Methods

MICE

All animals were maintained with appropriate care according to the United Kingdom Animal Scientific Procedures Act 1986 and the ethics guidelines of the Francis Crick Institute.

Derivation of mouse ESC from blastocysts

XY Mouse ESC (TESCO-CFP; R26-rtTA, clone 7) were derived from the inner cell mass of blastocyst-staged embryos. TESCO-CFP heterozygous mice were bred with R26-M2rtTA homozygous mice (generated from Jax mouse line 016836- R26-M2rtTA; TetOP-H2B-GFP). E4.5 Blastocyst embryos were flushed in M2 media (M7167, Sigma) from the uterus and each embryo was placed on a 0.1% gelatin coated 24-well dish containing NDiff+2i/LIF media (described below). 7-14 days following seeding, a colony was formed and expanded. Cells were genotyped for sex chromosomes (*74*), TESCO-CFP (*75*) and the R26-M2rtTA allele (Jax protocol https://www.jax.org/strain/016836). Primers used for genotyping are depicted in Supp Table S1. Most of the differentiation work was carried using clone 7 which was XY, TESCO-CFP; R26-M2rtTA.

Mouse ESC culture

TESCO-CFP; R26-M2rtTA; XY ESCs were routinely cultured on 0.1% Gelatin (G9391, Sigma) coated dish with 2i/LIF media [NDiff medium (Y40002, Takara) supplemented with Penicillin-Streptomycin (P/S-15140-122, Invitrogen), L-Glutamine (L-Glu-25030-024, Invitrogen), Beta-mercaptoethanol (M6250, Sigma), 1 µM of PD0325901 (1408, Axon), 3 µM of CHIR99021 (1386, Axon), and 1000 U/ml of LIF (ESG1107, Millipore)].

Intermediate mesoderm differentiation of mouse ESCs

For differentiation, ESC cultured on 2i/LIF were seeded for 1 passage on dishes coated with Gel-MEF: 0.1% gelatin coating for 15 minutes followed by coating with MEF media for at least 2 hours [DMEM, 10% FCS, P/S, L-Glu]. ESCs were seeded into KSR media [Ad-DMEM/F12 (12634, Life technologies), 20% knock out serum replacement (10828010, Life technologies), P/S, L-Glu, Beta-mercaptoethanol and 1000 U/ml of LIF].

For epiblast-like cells (EpiLCs) differentiation, 2.5x10^5 cells were seeded on Gel-MEF coated 6-well dish wells. EpiLC differentiation was performed as previously described (*50*). Briefly, EpiLCs were induced by culturing ESC in N2B27 minus Vitamin A (12587-010, Life technologies) medium containing 20 ng/ml activin A (338-AC, R&D), 12 ng/ml bFGF (233-FB, R&D) and 1% knockout serum replacement (KSR, 10828010, Life technologies) for 2.5 days (~60 hours).

Early mesoderm differentiation was induced by growing the cells as previously described (*51, 69*) in a chemically defined medium (CDM) containing PVA (P8136, Sigma) and supplemented with 20 ng/ml bFGF, 10 μM LY294002 (1130, TOCRIS) and 10 ng/ml BMP4 (314-BP, R&D), named FLyB for 36 hours.

Intermediate mesoderm differentiation was performed for 3.5 days in CDM-PVA media supplemented with the following growth factors as described in the result section (IM1-5): FGF (5ng/ml), BMP4 (20ng/ml), RA (100nM, R2625, Sigma), CHIR99021 (0.3 μM), activin A (10ng/ml). IM3 which showed the best results contained FGF (5ng/ml), BMP4 (20ng/ml) and RA (100nM).

IM1: FGF (5ng/ml) + BMP (20ng/ml)

IM2: FGF (5ng/ml) + BMP (20ng/ml) + RA (100nM) + A (10ng/ml)

IM3: FGF (5ng/ml) + BMP (20ng/ml) + RA (100nM)

IM4: FGF (5ng/ml) + BMP (20ng/ml) + RA (100nM) + CHIR (0.3 μM)

IM5: FGF (5ng/ml) + BMP (20ng/ml) + RA (100nM) + A (10ng/ml) + CHIR (0.3 μM)

Following viral induction (described below), IM cells were cultured in a **"Sertoli media"** containing Ad-DMEM/F12 supplemented with P/S, L-Glu, HEPES, X1 B27 (GIBCO 17504-044) and nAC (Sigma A9165, 1.25 mM). The following growth factors were added: mEGF (Invitrogen PMG8043, 50 ng/ml), rhFSH (R&D 5925-FS, 22 ng/ml), rmFGF9 (R&D 7399-F9, 50 ng/ml), Prostaglandin D2 (Cayman 12010, 500 ng/ml), Testosterone (Sigma T1500, 1 μM) and Activin A (R&D 338-AC, 50 ng/ml).

Lentivirus production

Lentiviruses encoding Nr5a1 and Dmrt1 were generated by transfecting HEK293T cells with the plasmids FUW-TetO-Nr5a1/Sf1 (Addgene 41081, gift from Y. Buganim) or FUW-TetO-Dmrt1 (Addgene 41083, gift from Y. Buganim) along with the packaging vectors pMD2.G (Addgene

12259) and psPAX2 (Addgene 12260) using XtremeHP transfection reagent (6366236001, Roche). A day after transfection media was replaced to minimal volume of IM3 media and viral particles were collected 48 hours post transfection. Supernatant containing viral particles was filtered through 0.45 µm filter. Viral particles were supplemented with 2 µg/ml of polybrene (Sigma, H9268) and were used for infection of IM cells.

Following 3.5 days of IM differentiation, each 6 well was split into 2 new wells and viruses produced in IM media were added onto the newly seeded cells. Viruses were left on the cells for 24 hr after which media was changed to "Sertoli media" supplemented with 2 µg/ml Doxycycline (D9891, Sigma).

Flow cytometry of mouse differentiated CFP positive cells

CFP expressing early gonadal progenitors were FACS sorted 4 days following Dox addition. Cells were prepared into single cell suspension using trypsinization and filtered via a 30 µm filter (CellTrics, 04-004-2326). Sorting was performed on the BD *FACSARIA*™ III cell sorter using a 450 laser. CFP negative IM3 cell sample was used to set up the gating.

RNA isolation, cDNA preparation and Quantitative Real-Time Polymerase chain reaction (qRT-PCR) for mouse differentiated cells

Total RNA was extracted using Tri Reagent (Sigma, T9424) according to the manufacturer's protocol. RNA yield was quantified using a NanoDrop spectrophotometer (NanoDrop Technologies), and 1500 ng RNA was treated with RQ1 DNase (Promega) and used to synthesize cDNA using the SuperScript™ III Reverse Transcriptase kit (Invitrogen). qRT-PCR reactions were performed in duplicate using SYBR Green PCR master mix (Invitrogen) and 150 nM each of forward and reverse primers and analysed on the Applied Biosystems 7500 Real-Time PCR System (Thermo Fischer Scientific). Primers are listed in Supp Table S2. Relative mRNA levels were determined by calculating $2^{-\Delta\Delta Ct}$ values relative to the normalizer gene *Pbgd*. Relative gene expression is presented as the mean $2^{-\Delta\Delta Ct}$ values (error bars are SEM of the $2^{-\Delta\Delta Ct}$) for triple biological repeats. Statistical analysis was performed using one-way ANOVA on the $2^{-\Delta\Delta Ct}$ values using the Prism 9 software (GraphPad) (* < 0.05, ** < 0.01, *** < 0.001, **** < 0.0001).

RNA-Sequencing

Total RNA was extracted Tri Reagent as described above. Triplicate of RNA samples of ESC, EpiSC-like cells, M-like cells, IM-like cells (IM1-5) and CFP sorted Sertoli-like cells were used for RNA-Seq. RNA quality and quantity was analysed on the Qubit (Thermo Fischer Scientific) and Tapestation (Agilent). Libraries were prepared using the TrueSeq Library Prep kit V2 (Illumina) according to the manufacturer's instructions. Sequencing was performed on the Illumina HiSeq 4000 system (Paired end, 75 bp).

Bioinformatic analysis

The mouse ESC differentiation RNA-Seq data has been deposited to the GEO under the accession number: GSE165133.

Data collection

Published RNA-Seq data from mouse embryonic gonad tissue relating to stages E10.5, E11.5, E12.5 and E13.5 were downloaded from the NCBI's Short Read Archive under accession: SRP076584 (*16*). Published RNA-Seq data from sorted TESCO-CFP E15.5 Sertoli cells was downloaded from the NCBI's Short Read Archive under accession: SRP033562 (*53*). Single-cell RNA-seq on embryonic mouse gonads was downloaded from the GEO under accession: GSE97519 (*15*).

Alignments and abundance estimation

Cutadapt v1.9.1 was used to trim adapter sequences from reads with the following options: -a AGATCGGAAGAGC –A AGATCGGAAGAGC -e 0.1 –q 10. Gene-level abundance estimates were calculated with RSEM v1.3.0 (*71*), using STAR v2.5.2a (*70*) to align reads against the GRCm38 genome assembly with Ensembl release 86 transcript annotations. A copy of the code used for this step (*72*) is freely available as a Nextflow pipeline and is available at Zenodo (https://zenodo.org/record/4270402#.Y3zrhOzP3a0) and the Github repository (https://github.com/crickbabs/BABS-RNASeq.git).

Data exploration and differential expression

Gene-level RSEM estimated counts and average transcripts lengths were imported into R 3.6.0 using the tximport function from the tximport package (*76*). These were used to create a

DESeqDataSet object for further analysis using the Bioconductor package DESeq2 (*72*). Data were normalised for differing library size using DESeq2's default method, leveraging the average transcript length information. Differential gene expression analysis between replicate groups was assessed using the default Wald test. Genes were called significant it they passed a combined filter of i) FDR<=0.01, ii) fold change >= +/-2, iii) base-mean >= 100 from the Wald test results and iv) a mean normalised read count of >= 100 in at least one of the tested replicate groups.

Principal Components Analysis (PCA) was used to assess the relationship between gene expression across samples using the PCAtools package's "pca" function (center=TRUE, scale=FALSE, removeVar=0.9). Data were first variance stabilised using DESeq2's "vst" function, which is roughly similar to putting the data on the log2 scale, while also dealing with the sampling variability of low counts. Only the top 10% most variant genes across selected samples were used to generate the visualisations.

While majority of the samples were generated *in-vitro* there are a number that were generated *in-vivo*, specifically: E10.5, E11.5, E12.5, E13.5 gonads and E15.5 sorted Sertoli cells. This difference in protocol appeared to dominate the first principal component (PC1, 47.47% variation) when combining both *in-vivo* and *in-vitro* data. A bi-plot of PC2 and PC3 better reflected the expected biology.

The *in-vitro/in-vivo* protocol specific effect was modelled and removed from the variance stabilised data using the limma package's "removeBatchEffect" function for the purposes of visualisation of combined *in-vitro* and *in-vivo* samples only. PCA analysis of the corrected data showed that PC1 was analogous to PC2 of the uncorrected data.

Sample similarity was assessed using a Poisson dissimilarity matrix constructed from the un-corrected normalised counts of all samples using the "PoissonDistance" function from the PoiClaClu package. All genes with a count greater than 0 in at least a single sample were included in the generation of the matrix unless otherwise stated.

Heatmaps were generated using the variance stabilised data. Data were additionally scaled per gene using a z-score to aid visualisation. Columns (samples) and rows (genes) were each hierarchically clustered using a "complete" clustering method on a set of Euclidean distances.

Exploration of the genes driving the change using differential expression analysis

Differential expression analysis between the *in vitro* reprogramming cells were performed using DEseq2 with the "LRT" test on library size normalised read counts. Genes presenting an adjusted p-value < 0.05 were plotted as a heatmap using the Pretty Heatmap R package and clustered into 15 profiles using "ward.D2" algorithm (P1-15, Supplementary Data S2-3). Genes with roughly similar expression profiles were merged and subjected to a GO term enrichment analysis using ClusterProfiler (Biological processes). GO term similarity were reduced using the similarity function from ClusterProfiler to remove redundant information (Supplementary Data S4-6).

Comparison to scRNA-seq gonadal data

Single-cell RNA-seq data from Stévant et al. (*15*) (GSE97519) were mapped on the mouse reference genome (GRCm38) using GemTools, duplicated reads and non-uniquely mapped reads were discarded with Samtools, and gene expression was assessed using an in-house pipeline as described in Stévant et al. (*15*).

Analysis of the Stévant et al. data and cell clustering

Stévant et al. Single cell dataset is composed of 435 NR5A1-eGFP sorted cells from XY mouse embryos from five different developmental stages (E10.5, E11.5, E12.5, E13.5, and E16.5).

As described in the original paper, the cells are classified into 6 populations using unsupervised clustering (**C1-C6**, Supplementary Figure 7A-B): the E10.5 and E11.5 early progenitor cluster (**C2: Early Prog.**) expressing progenitor-related genes such as *Nr2f2, Tcf21* (*Pod1*) and *Lhx9*; the interstitial progenitors cluster (**C3: Int. Prog**) with cells from E12.5 onward and expressing *Nr2f2*, *Arx* and *Pdgfra*; the pre-Sertoli (**C4: Pre-Sertoli**) and Sertoli (**C6: Sertoli cells**) clusters with the E11.5 *Sry* expressing cells and the E12.5 onward differentiated Sertoli cells respectively; the fetal Leydig cell cluster (**C5: Leydig cells**); and the endothelial cluster (**C1: Endothelial cells**). These cells were probably captured by chance during the single-cell isolation step. The time-series single-cell data allowed to reconstruct *in silico* the NR5A1+ cell lineage during XY fetal gonad differentiation (Supplementary Figure 7C). The Sertoli cells (**C4** and **C6**) differentiate from the early progenitor cell population (**C2**) during a narrow window of time around E11.5. Passing this time window, the remaining progenitor cells reside in the testicular *interstitium* compartment (**C3**) and operate gradual transcriptomic changes that restrict their fate to steroidogenic precursors that eventually give rise to fetal Leydig cells (**C5**).

Generation of pseudobulks

To be able to compare bulk RNA-seq with the single-cell RNA-seq data from Stévant et al. (*15*), we generated pseudobulks per cell types by summing the read count per genes for each of the six cell clusters identified in the original study. For each dataset, we selected the protein coding genes from the read count per gene matrices. For each comparison, the compared dataset read counts were normalised using DEseq2 size factor. To compare the samples, we computed pairwise Spearman correlations and generated a heatmap using Pretty Heatmap R package (default clustering parameters). Because the single-cell data show a strong batch effect comparing to bulk RNA-seq, we thought to repeat our analysis on the genes constituting the cell type identity rather than the whole transcriptome. For that, we extracted the genes that are over-expressed in each of the cell clusters present in the Stévant et al. (*15*) study (C1-C6) (Supplementary Data S7) with a strict threshold of adjusted p-value < 0.001. We selected these genes in each of the RNA-seq datasets and re-ran the Spearman pairwise correlation analyses.

Immunofluorescence staining for mouse differentiated cells

Immuno staining was performed onto glass chamber slides. Cells were fixed for 10 min in 4% PFA (Sigma, P6148) at RT. Blocking was performed in 5% Donkey serum (Sigma, D9663) in PBS + 0.1% Triton (PBST) solution for 1 hr at RT. Primary antibodies (listed in Table S3) were incubated in PBST supplemented with 1% Donkey serum O/N at 4ºC. 3 washes in PBST were performed and secondary antibodies were incubated in PBST supplemented with 1% Donkey serum for 1 hr at RT. Secondary antibodies used were Donkey anti-rabbit/goat Alexa Fluor 568 (1:500, Invitrogen) and Donkey anti-mouse Alexa Fluor 647 (1:500, Invitrogen). All immunofluorescence slides were also stained with 4',6-diamidino-2-phenylindole (DAPI, Molecular Probes), to visualize nuclear DNA. Images were taken on the Nikon ECLIPSE Ti2-U fluorescent microscope.

HUMAN

Human induced Pluripotent Stem Cell (hiPSC) lines

Peripheral blood was collected from two individuals. First, a 46,XY girl carrying a *de novo* heterozygous p.Arg313Cys pathogenic variant in *NR5A1*, identified by exome sequencing that causes a complete lack of testis determination (46,XY complete gonadal dysgenesis, (*56*)). The

second sample was from her brother, a healthy 46,XY male, who did not carry the *NR5A1* variant. Peripheral blood mononuclear cells (PBMC) from these two samples were reprogrammed into induced pluripotent stem cells (Phenocell, Grasse). Briefly, PBMCs were cultured in complete StemSpan™ SFEM II medium (#09605; StemCell Technologies) were reprogrammed with the Epi5™ Episomal iPSC Reprogramming Kit (#A15960, ThermoFisher Scientific) according to manufacturer's instructions. The reprogramming vectors were introduced by nucleofection and the transfected cells were harvested and plated on Laminin 521-coated P6 culture dish in ReproTeSR™ medium (#05926; StemCell Technologies). Medium was changed daily until iPSC colonies appeared (10-12 days), then diluted 1/1 with mTEeSR™1 (#85850; StemCell Technologies) until colonies were large enough to pick and expand. Colonies are selected on morphological criteria, isolated, and further amplified in mTEeSR™1 over 10-12 passages to perform quality control by Karyotyping, Array CGH and exome sequencing. A third iPSC line was bought from Phenocell. Briefly, this line was reprogrammed from PBMC obtained from an 46,XX female donor. A fourth line of iPSC was generated after correction of the *NR5A1* mutation, using CRISPR/CAS9, in the iPSCs derived from 46,XY girl (CELIS platform, Institut du Cerveau et de la Moëlle, ICM, la Pitié-Salpétriere, PARIS).

Culture of hiPSC

hiPSCs were cultivated in feeder-free mTeSR™Plus medium (#100-0276, StemCell Technologies) on qualified "human Embryonic StemCell" (hESC) matrigel (#354277, Corning). Thiazovivin was added for 48 hours to the medium to prevent spontaneous differentiation (#130-106-542, Miltenyi Biotec) at thawing and passaging. When the colonies reached 70-80% of confluence, cells were split into clumps or aggregates with the ReLeSR (#05872, StemCell Technologies) medium and, depending on the confluence, clumps were diluted at 1:10 or 1:20 into a new freshly Matrigel coated flask.

Differentiation of hiPSC

24 hours before differentiation, colonies of iPSC (80% of confluence) were dissociated into single cells solution, using the Gentle Cell Dissociation reagent (#07174, StemCell Technologies), and seeded on Matrigel coated flasks and plates (T25cm^2 flask, 6-well plate and chamber slide) with a high density of cells (1:2 dilution). Similar to murine pluripotent cells, hiPSCs were subjected to

serial differentiation in conditioned medium with minor modifications of the medium composition. The basal medium used for subsequent steps was Chemically Defined Medium (CDM-PVA) containing 250ml of advanced DMEM (#31331028, ThermoFisher Scientific), 250ml of Iscove's Modified Dulbecco's Media (IMDM, #31980030, ThermoFisher Scientific), 0.1% of cold water soluble polyvynilic alcohol (#P8136, MerckMillipore), 5ml of penicillin-streptomycin (#15140122, 10 000U/ml, ThermoFisher Scientific), 5ml of concentrated lipids (11905031, 1:100, ThermoFisher Scientific), monothioglycerol (MTG, 20ul, water miscible 0.1M, #M6145, MerckMillipore) and 300ul of transferrin (#1065220200, water soluble, MerckMillipore). For mesodermal induction, cells were incubated for 36 hours in FlyB medium (CDM-PVA with bFGF (20ng/ml; #233-FB, R&D), Ly294002 (10µM; Pi3K inhibitor, #L9908, MerckMillipore) and BMP (10ng/ml; #214-BP, R&D)). This was followed by directed differentiation towards intermediate mesoderm using IM3 medium, composed of CDM-PVA with bFGF (5ng/ml), BMP (20ng/ml) and retinoic acid (RA, 100nM; #R2625, MerckMillipore) for 48 hours, with a change of medium at 24 hours. To induce differentiation toward supporting cell lineages, the medium was changed to supporting Medium that is composed of 500ml of Advanced DMEM (#12634010, ThermoFisher Scientific), 5ml of Penicillin/ Streptomycin, 5ml of Insulin, Transferrin, Selenium (ITS, 100X #12097549, ThermoFisher Scientific) and 50ul of EGF (20 ng/mL; human recombinant #ab9697, Abcam). The differentiating cells were cultured in supporting medium, with the change of medium every 2-3 days until spontaneous tubular structures appear.

Flow cytometry of differentiated hiPSC

Once defined structures are visible the cells are dissociated into single cells. For cell sorting, $1x10^7$ cells were incubated with rabbit anti-CLAUDIN11 antibody (dilution 1:100, #36-4500, ThermoFisher Scientific) followed by anti-rabbit IgG associated with either Alexa 488 (#A11034, ThermoFisher Scientific) or 594 (#A11037, ThermoFisher Scientific). The stained cells were collected in FACS tube with cell strainer (#352235, Corning) and examined using MoFLO Astrios "Beckman Coulter" with Summit v62 (Beckman Coulter). After negative selection for Alexa 488 or 594 remaining gated cells were collected in Eppendorf tube containing 500µl of supporting medium. On an average CLAUDIN11 positive cells represented between 4-12% of the initial population depending on the differentiation efficiency. Sorted cells were seeded in a well of a 12

well-plate coated with non-qualified Matrigel (#354230, Corning), and cultured for several weeks to let the cells recover from the stress of sorting and grow.

Immunofluorescence staining for undifferentiated hiPSCs and differentiated cells

Undifferentiated hiPSC and differentiated cells were cultured on chamber-slides, and stained for OCT4/POU5F1, SOX9, FOXL2, CLAUDIN11, WT1, VIMENTIN and DMRT1 proteins according to the protocol described elsewhere (*77*). Briefly, after fixation in 4% PFA, permeabilization and blocking of non-specific epitopes, cells were incubated with the primary antibody (diluted in 3% BSA in PBS) in a humid chamber overnight at +4°C. The following dilutions of primary antibodies were used: anti-SOX9 (1:100, #14-9765-82, ThermoFisher Scientific), anti-OCT4 (1:100, #ab19857, Abcam), anti-FOXL2 (1:100, #ab5096, Abcam), anti-CLAUDIN11 (1:100, #36-4500, Life Technologies), anti-DMRT1 (1:100, a gift from Prof. David Zarkower. (Uni of Minesota) PMID: 23473982), WT1 (1:100, #Ab89901, Abcam), VIMENTIN (1:200, #MA5-11883, Thermofisher Scientific) and anti-SRY (1:100, #ab140309). After 16 h of incubation, cells were washed three times with 1xPBS for 5 min each. This was followed by incubation with the secondary antibody in 3% BSA for 1h at room temperature in the dark. Depending on the primary antibody, the following secondary antibodies were used- Goat anti-Rabbit IgG (H+L) Secondary Antibody, Alexa Fluor® 594 conjugate (1:1000, #A11037, Life Technologies), Goat anti-mouse IgG (H+L) Secondary Antibody, Alexa Fluor® 488 conjugate, (1:1000, #A11029 Life Technologies), Donkey anti-goat IgG (H+L) secondary antibody, Alexa Fluor® 488 conjugate, (1:1000, #ab150129, Abcam). Cells were washed three times with 1xPBS for 5 min each in the dark and incubated with DAPI diluted in PBS for 15min in the dark (1:2000, #62248, ThermoFisher Scientific). After three washes in PBS, slides were mounted using ProLong® Gold Antifade Mountant with DAPI (#P36931, ThermoFisher Scientific). Images were obtained with a Leica Microsystems DMI4000B microscope at 40x, 63x and 100X (with oil) magnifications. For the Suplementary figure images were acquired by confocal ZEISS LSM 800 microscope and 20X or 40X oil objectives with an optical sectioning in Z every 0.9 µm and a tile scan of 10 to 15 Z stacks. Image analyses were performed with Fiji software. Supplementary Movies 5-7 of reconstituted 3D images were realized using the 3D project plugging.

Quantitative Real-Time Polymerase chain reaction (qRT-PCR) for undifferentiated hiPSCs and derivatives

Total RNA was extracted from undifferentiated hiPSC and cells during the course of differentiation, using TRIzol reagent (#15596026, ThermoFisher Scientific). RNA yield was quantified with a NanoDrop spectrophotometer (NanoDrop Technologies), and 1000 ng RNA was used to synthesize complementary DNA (cDNA) with the Quantitect Reverse Transcription Kit (#205311, QIAGEN) as per manufacturer's recommendations. cDNA was diluted 1/25 prior to the qPCR. qPCR was performed using TaqMan Universal Master Mix II, with UNG (#4440038, Applied Biosystems) on a StepONEPlus qPCR machine (Applied Biosystems). The Following TaqMan probes (Applied Biosystems) were used; RPL19: #Hs02338565_gH; SOX9: #Hs01001343_g1; DMRT1: #Hs00232766_m1; NR5A1: #Hs00610436_m1; WT1: #Hs01103751_m1; FOXL2: #Hs00846401_s1; FGF9: #Hs00181829_m1; NANOG: #Hs02387400_g1; BRACHYURY/T: #Hs00610080_m1; NR2F2: #Hs00819630_m1; OSR1: #Hs01586544_m1; GATA4: #Hs0171403_m1; RSPO1: #Hs00543475_m; SRY: #Hs00976796_s1; MESP1 #Hs00251489_m1; PAX2 #Hs01057416_m1; LHX1 #Hs00232144_m1; AMH #Hs00174915_m1; HSD3β1 #Hs04194787_g1; STAR # Hs00986559_g1. Relative mRNA levels were determined by calculating $2^{-\Delta\Delta Ct}$ values relative to the 18S rRNA normalizer gene (RPL19). Relative gene expression is presented as the mean $2^{-\Delta\Delta Ct}$ values (error bars are SEM of the $2^{-\Delta\Delta Ct}$).

Statistics

Statistical analyses were carried out using GraphPad Prism 9 software (GraphPad). Quantitative data were subjected to a one-way ANOVA (* < 0.05, ** < 0.01, *** < 0.001, ns= not significant). followed by Bonferroni comparison.

Measurement of AMH concentrations

AMH (Anti-Müllerian Hormone) was measured by a one-step sandwich enzyme-linked immunosorbent assay (Access AMH, Beckman Coulter Company, Marseille, France (*73*)). AMH is sandwiched between two anti-AMH monoclonal antibodies, one conjugated to alkaline phosphatase, the other coated with paramagnetic particles. A Lumi-Phos 530 chemiluminescent developer was used to read the light output proportional to the concentration of AMH in the sample

relative to recombinant human AMH that was used as standard. The intra-assay coefficients of variation range from 1.41% to 3.3% and the inter-assay coefficients of variation from 3.04% to 5.76%.

Soft Matrigel substrates

Organoid grade Matrigel (#354263, Corning) was diluted to 50% (v/v) in supporting medium. 50-µl domes of Matrigel were cast onto a well of chilled 12-well plate and left for gelation for 30 min at 37°C. 50µl of concentrated cells ($1x10^6$ cells/ml) were pippeted onto the domes slowly and incubated at 37°C for 30min, after which 50µl of supporting medium was added carefully, and the plates were incubated for 48-72 hours. Cells self-aggregate on the top of the dome and make 3D structure and excess cells grow as a layer at the bottom of the dome. Images were obtained with a Leica Microsystems DMI4000B microscope at 40x, 63x and 100X (with oil) magnifications.

GONAChip microfluidic device and migration assay

GONAChip was composed of three channels (one central for the Matrigel flanked by two media channels) with gaps in the walls separating the channel in order to ensure a proper medium gel/medium interface. The proper confinement of the Matrigel in the central channel was ensured by hydrophobic pinning. Chips were produced by means of photo- and soft-lithography as described in Jeon *et al.* (*78*). Briefly a master mould of 160 um in hight was created by means of photolithography. Replication of the device were performed with poly-dimethyl-siloxane (PDMS, Silgard 184; Dow Chemical). Finally, we used oxygen plasma to bond the PDMS slabs onto #1.5 Glass coverslips. Prior to use we performed a 20 min sterilization step in a UVO cleaner (Jelight, CA, US). Organ grade Matrigel (#354263, Corning) was diluted with advanced DMEM medium to have 50% (v/v) Matrigel (50µl of medium into 50µl of Matrigel). The central channel was filled with 1.3µl of this solution. After gelation at 37°C in the incubator, media channels were filled with supporting medium. Confluent sorted cells were detached, counted, and concentrated at $1x10^6$ cells/ml. Few µl of cell suspension was introduced in each chip. Chips were flipped to 90° to allow cells to sediment on the Matrigel channel side. After washing the chips were left in standard cell culture incubator (37°C, 5% CO2). When performing Time-lapse imaging, seeded GONAChips were placed under an inverted microscope equipped with a temperature, humidity, and CO2

control system. (Inverted Z1 Axio Observer, ZEISS). Pictures were taken every 15 min for 70 hours. Images are processed using the ZENlite Software (ZEISS) to extract videos and images.

Scratch-wound assay

Differentiated cells derived from iPSCs from 46,XY male, 46,XY DSD patient and 46,XY DSD cell line with CRISPR/CAS9 correction of the pathogenic variant (46,XY DSD-EDIT) were plated at the density of 50 × 105 cells per well in a 96-Well ImageLock microplate (Sartorius Cat. No. #4379). 24 hours post seeding, in the confluent wells, a thin wound was created by scratching the culture with Incucyte® 96-Well WoundMaker Tool (Sartorius Cat. No. 4563). Images of the wound closure were acquired on the hour, for 24 hours at 10X magnification using the IncuCyte® SX5 Live-Cell Analysis Instrument (Sartorius, Germany). The wound closure was measured by calculating the decrease of the wound width over time using Incucyte® Scratch Wound Analysis Software Module (Sartorius Cat. No. 9600-0012). The slope of the line generated in the different conditions was estimated using a curve fitter online tool (http://statpages.info/nonlin.html). The significance of the difference between the slopes was calculated using (A) an online statistic calculator (http://www.danielsoper.com/statcalc) and (B) Wilcoxon Signed-Rank Test Calculator (https://www.aatbio.com/tools/mann-whitney-wilcoxon-signed-rank-test-calculator).

Correction of *NR5A1* p.Arg313Cys by CRISPR/CAS9

$1x10^6$ hiPSCs were nucleofected with RNP complex (225 pmol of each RNA crRNA; #Alt-R® CRISPR-CAS9 crRNA, IDT; GCTGGACCTGGC**a**GTAGATG (The target site in the sequence is specified by the small bold letter), tracrRNA-ATTO+; #Alt-R® CRISPR-CAS9 tracrRNA, IDT) and 120 pmol of Cas9 protein; #Alt-R® S.P. Hifi CAS9 nucelase 3 NLS, IDT; #Alt-R® CAS9 electroporation enhancer, IDT) and HDR template (500 pmol ssODN; TGCCCGGTGACCAGCAGGATGCTGCCCTCCTTGCCGTGCTGGACCTGGC**g**GTAGATG TGATCGAACACCAGCAGCTCGCTCCAGCAGTTCTGCAGCAGCG (The inserted change in the sequence is specified by the small bold letter); Ultramer DNA oligo (ssODN repair template), IDT). 24hrs later, ATTO+ transfected iPSCs were sorted by FACS and plated at very low density (10 cell/cm^2) on Ln521 (#77003, StemCell Technologies) with CloneR supplement (#05888, StemCell Technologies) for clonal selection. One week later, hiPSC clones were picked under a stereomicroscope and cultured on Ln521 in 96 well plates. When confluent, iPSC clones were

duplicated for cryopreservation and DNA extraction. Clones were then analysed by PCR (iCS-digital^{TM} PSC – 24 probes) to assess the genomic integrity of the stem cell lines both before and after correction by CRISPR/CAS9 modification (Stem Genomics, Montpellier, France).

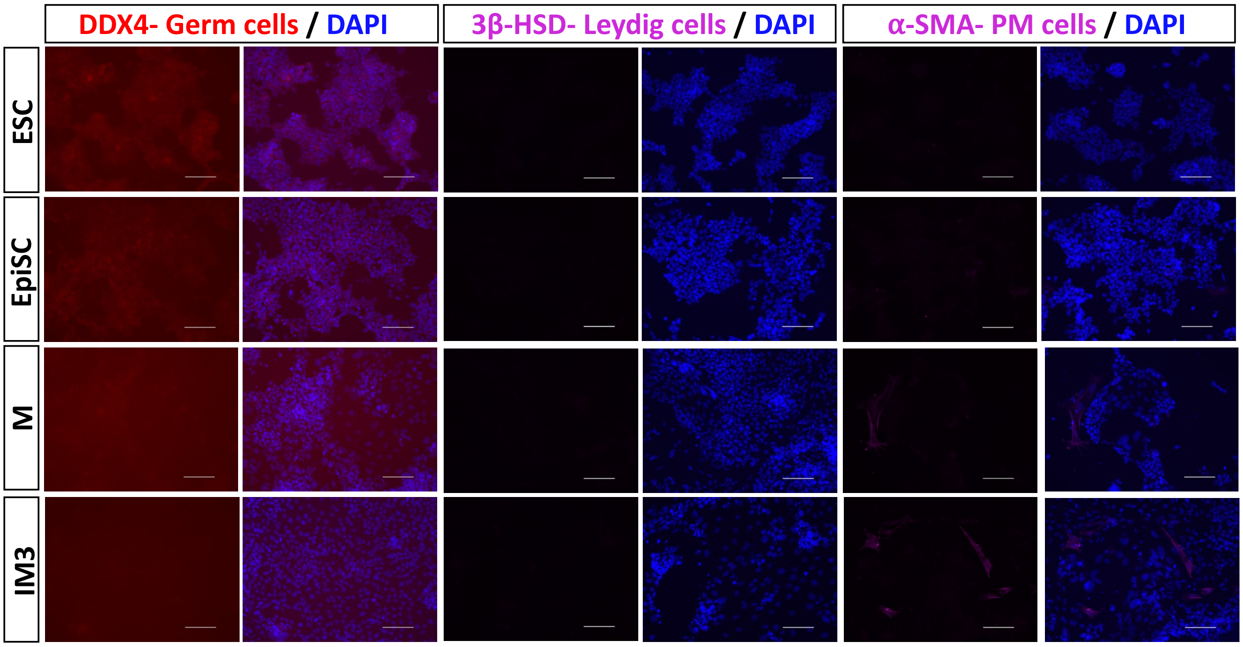

Supplementary Figure 1. Immunostaining of cells undergoing differentiation (ESC, EpiSC, M, IM3) using gonadal markers. Staining was done using the DDX4 (Germ cells, Red), 3β-HSD (Leydig cells, Magenta) and αSMA (Peritubular myoid cells, Magenta) antibodies. The stained protein is depicted at the top bar. Left panel of each bar is the protein staining alone, right panel is merge with DAPI. Scale bars are 100 µm.

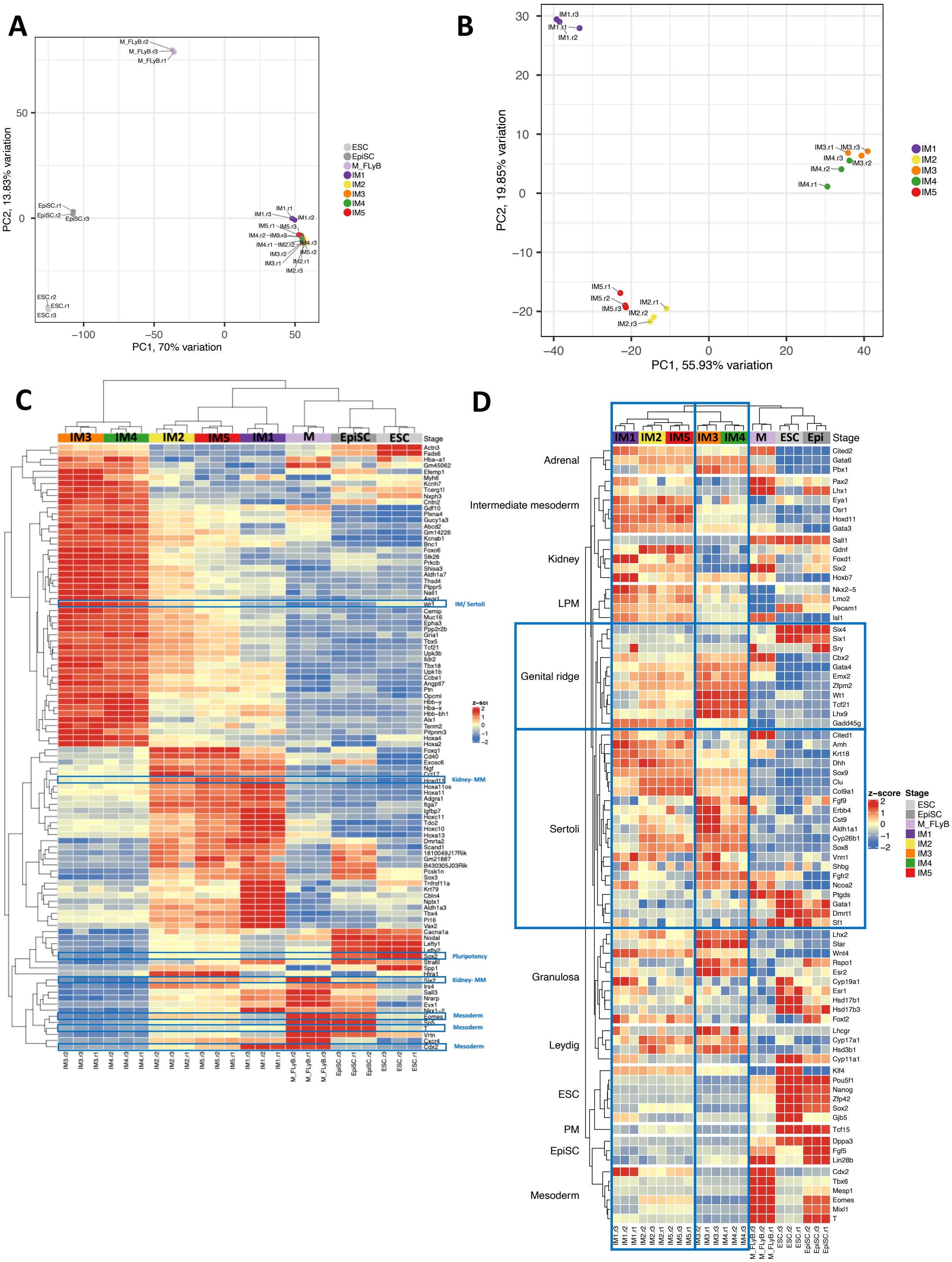

A
PC2, 13.83% variation
PC1, 70% variation
ESC
EpiSC
M_FLyB
IM1
IM2
IM3
IM4
IM5
B
PC2, 19.85% variation
PC1, 55.93% variation
C
IM3
IM4
IM2
IM5
IM1
M
EpiSC
ESC
Stage
IM/ Sertoli
Kidney- MM
Pluripotency
Kidney- MM
Mesoderm
Mesoderm
Mesoderm
D
IM1
IM2
IM5
IM3
IM4
M
ESC
Epi
Stage
Adrenal
Intermediate mesoderm
Kidney
LPM
Genital ridge
Sertoli
Granulosa
Leydig
ESC
PM
EpiSC
Mesoderm
Cited2
Gata6
Pbx1
Pax2
Lhx1
Eya1
Osr1
Hoxd11
Gata3
Sall1
Gdnf
Foxd1
Six2
Hoxb7
Nkx2-5
Lmo2
Pecam1
Isl1
Six4
Six1
Sry
Cbx2
Gata4
Emx2
Zfpm2
Wt1
Tcf21
Lhx9
Gadd45g
Cited1
Amh
Krt18
Dhh
Sox9
Clu
Col9a1
Fgf9
Erbb4
Cst9
Aldh1a1
Cyp26b1
Sox8
Vnn1
Shbg
Fgfr2
Ncoa2
Ptgds
Gata1
Dmrt1
Sf1
Lhx2
Star
Wnt4
Rspo1
Esr2
Cyp19a1
Esr1
Hsd17b1
Hsd17b3
Foxl2
Lhcgr
Cyp17a1
Hsd3b1
Cyp11a1
Klf4
Pou5f1
Nanog
Zfp42
Sox2
Gjb5
Tcf15
Dppa3
Fgf5
Lin28b
Cdx2
Tbx6
Mesp1
Eomes
Mixl1
T
z-score
Stage
ESC
EpiSC
M_FLyB
IM1
IM2
IM3
IM4
IM5

Supplementary Figure 2. Transcriptomic analysis of the *in vitro*-differentiated cells (A) Principal component analysis (PCA) of selected *in vitro* differentiated samples based on normalised mRNA expression level after batch correction. The top two PCs are shown. 70% of the variation is explained by PC1 and separates intermediate mesoderm samples from the other differentiation time points analysed. Three biological replicates were analysed for each sample type. (B) Principal component analysis (PCA) of the five different IM samples (IM 1-5) based on normalised mRNA expression level after batch correction. The top two PCs are shown. (C) Heatmap of the 100 most differentially expressed genes (50 most upregulated / 50 most down regulated) between IM1/2/5 and IM3/4. Markers of the various lineages are depicted by blue rectangles and the tissue they represent is denoted on the right. Three biological replicates were analysed for each sample type. (D) Heatmap of selected genes which represent known markers of the following lineages: Embryonic stem cells (ESC), Epiblast stem cells (EpiSC), Mesoderm (M), Intermediate mesoderm, Lateral plate mesoderm (LPM), Paraxial mesoderm (PM), Genital ridge, Sertoli cells, Leydig cells, Granulosa cells, Kidney and Adrenal. Three biological replicates were analysed for each sample type.

A

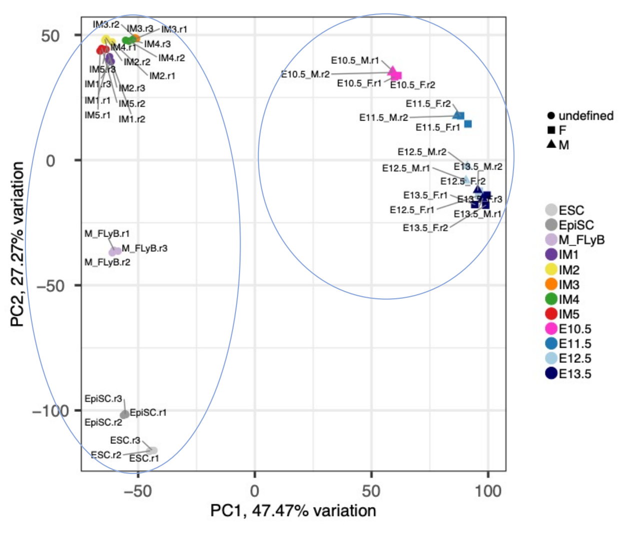
PC1, 47.47% variation
PC2, 27.27% variation
undefined
F
M
ESC
EpiSC
M_FLyB
IM1
IM2
IM3
IM4
IM5
E10.5
E11.5
E12.5
E13.5

B

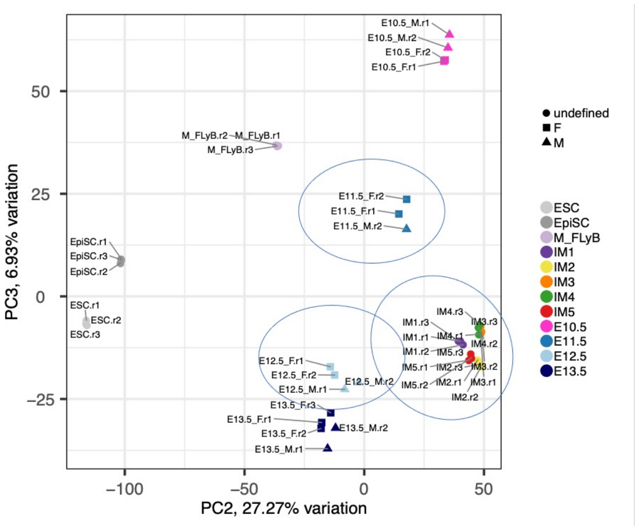
PC2, 27.27% variation
PC3, 6.93% variation
undefined
F
M
ESC
EpiSC
M_FLyB
IM1
IM2
IM3
IM4
IM5
E10.5
E11.5
E12.5
E13.5

C

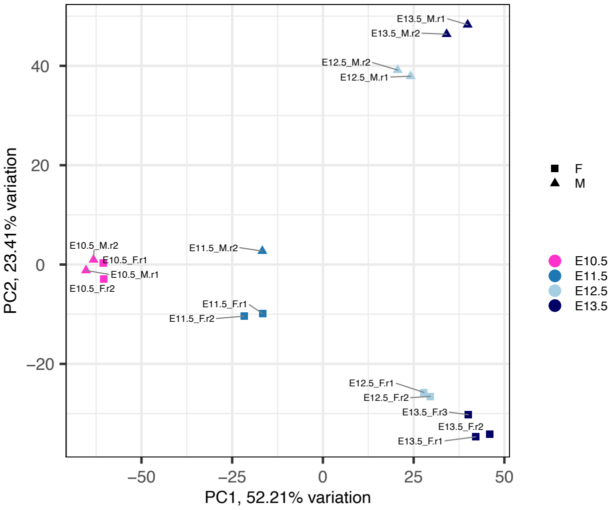
PC1, 52.21% variation
PC2, 23.41% variation
F
M
E10.5
E11.5
E12.5
E13.5

Supplementary Figure 3. Transcriptomic analysis comparison of the *in vitro*-differentiated cells and *in vivo* bulk gonadal cells (A) Principal component analysis of selected *in vitro* differentiated and *in vivo* gonad samples *(16)* based on normalised mRNA expression level without batch correction. The top two PCs are shown. Three biological replicates were analysed for each sample type. Without data correction the PC1/PC2 separates between the *in vitro*-derived (left side) and *in vivo*-derived (right side) samples. (B) PC2/PC3 Principal component analysis allows the temporal positional determination of IM *in vitro* derived cells within the *in vivo* samples analysed. (C) Principal component analysis of the Zhao et al., *(16) in vivo* gonadal data from XY and XX E10.5-E13.5 gonads. From E12.5 onwards there is a clear separation between XY and XX samples.

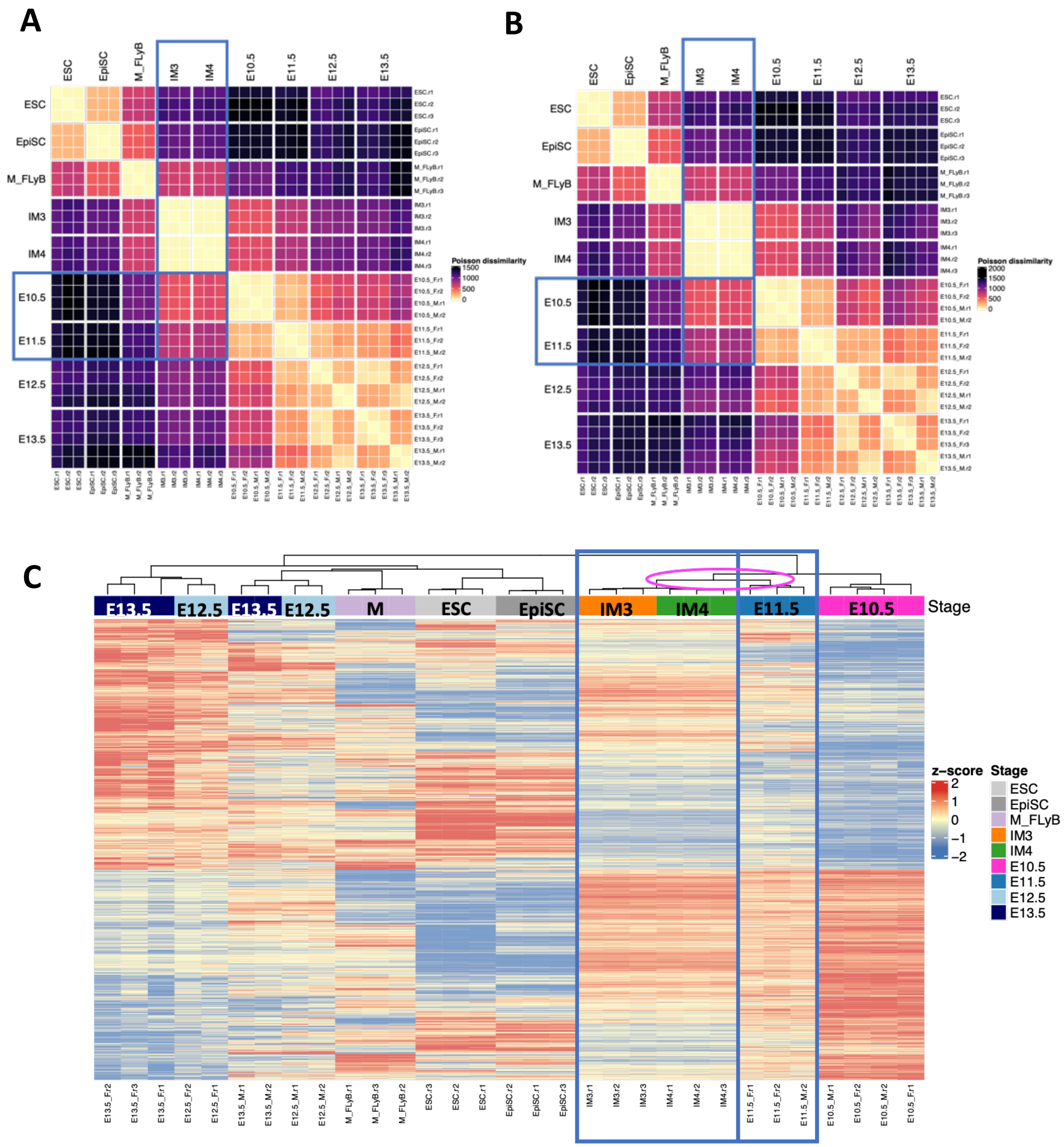

Supplementary Figure 4. Transcriptomic comparison of *in vitro*- and *in vivo*-derived gonadal cells (A) Heatmap of Poisson dissimilarity scores showing transcriptional similarities between samples. Dissimilarity scores were calculated from a normalised abundance matrix of genes differentially expressed between E10.5 XY and E13.5 XY gonads *(16)* Blue boxes indicate the similarity between IM3/4 and E10.5-E11.5 gonadal cells. Dark purple denotes dissimilarity while bright yellow denotes high similarity. (B) Heatmap of Poisson dissimilarity scores showing transcriptional similarities between samples. Dissimilarity scores were calculated from a normalised abundance matrix of genes differentially expressed between E10.5 XX and E13.5 XX

gonads *(16)*. Blue boxes indicate the similarity between IM3/4 and E10.5-E11.5 gonadal cells. (C) Heatmap of genes most differentially expressed between the E10.5 and E13.5 XX female gonads following batch correction *(16)*. IM3/IM4 cluster closely to the E11.5 *in vivo* gonadal cells. Gene-level expression across samples is shown as a z-score running from red (high) to blue (low). Three biological replicates were analysed for each sample type.

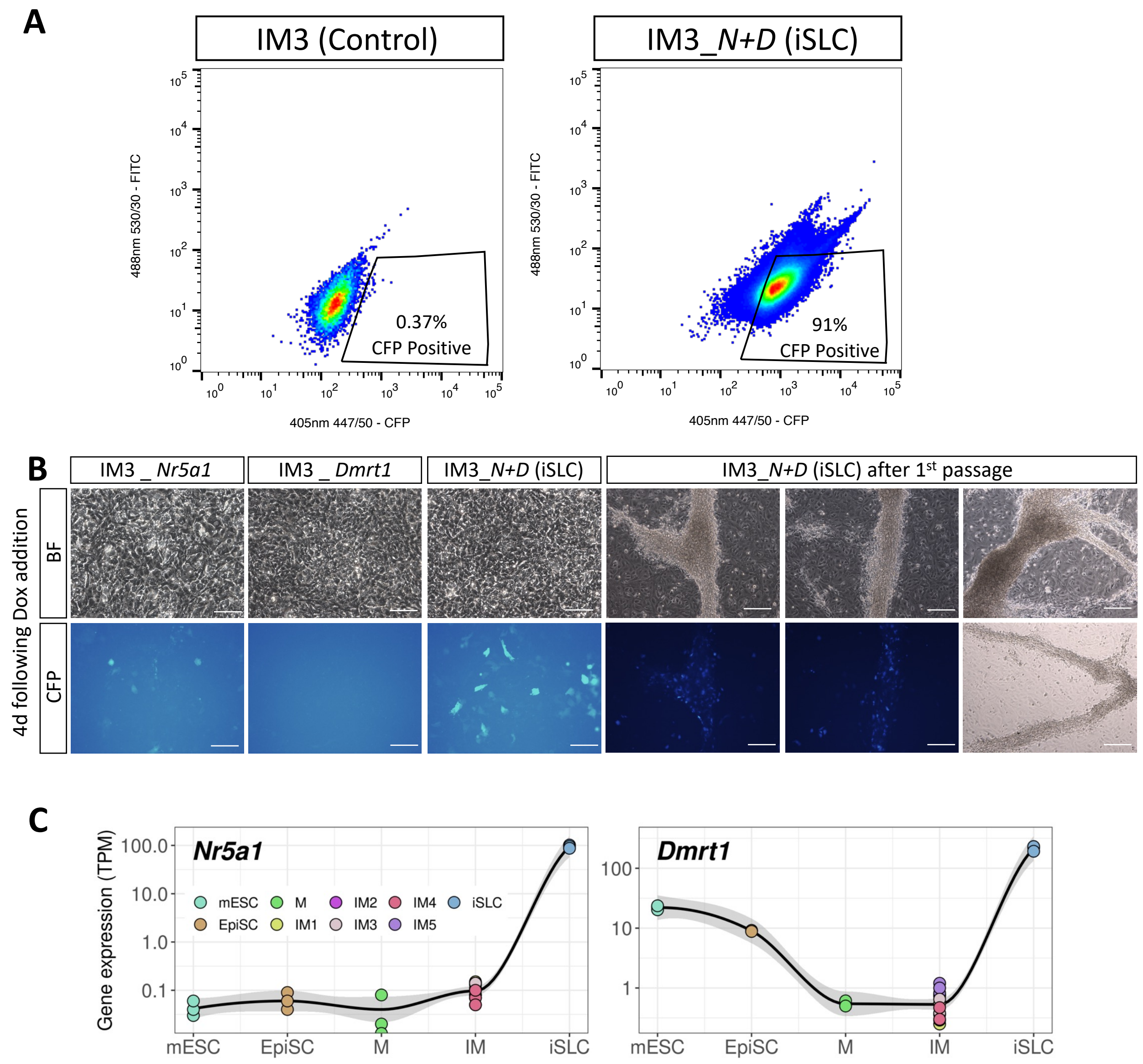

Supplementary Figure 5. Induction of iSLC using *Nr5a1* and *Dmrt1* over expression in IM3 cells. (A) Flow cytometry analysis showing CFP expression in IM3 differentiated cells without viruses (d13, left panel) and IM3 cells overexpressing *N* (*Nr5a1)* and D (*Dmrt1)* (iSLC) (d13, right panel). (B) Bright field and fluorescent images of IM3 cells overexpressing either *Nr5a1*, *Dmrt1* or both (*N*+*D*). Cells were imaged 4 days post Dox addition or after they were passaged (Bi), when tubule-like structures start to appear. Most CFP positive cells are located within tubules (Bii). (C) Gene expression analysis based on the bulk RNA-Seq (TPM presented) of the *Nr5a1* and *Dmrt1* genes. Both genes are strongly expressed at the iSLC stage.

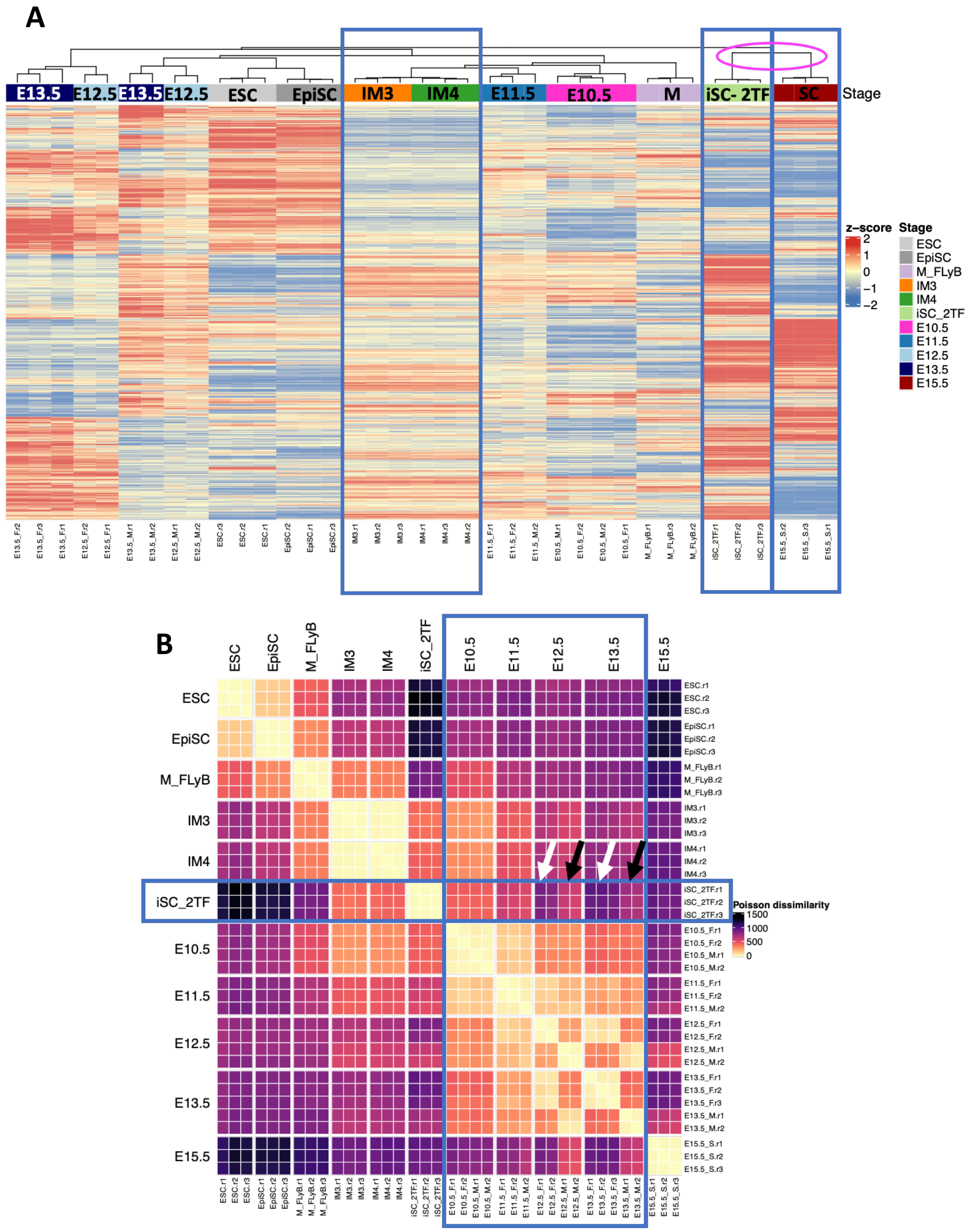

Supplementary Figure 6. Comparing the transcriptome of *in vitro*-derived gonadal cells with *in vivo* gonadal cells and E15.5 purified Sertoli cells (A) Heatmap of normalised abundance after batch correction for genes differentially expressed between E13.5 XY and E13.5 XX gonads. Induced Sertoli-like cells following forced expression of *Sf1* and *Dmrt1* (iSC-2TF/ iSLC) cluster closely to the E15.5 TESCO-CFP purified Sertoli cells, however the gene expression pattern indicates that the two are not identical. (B) Heatmap of Poisson dissimilarity scores showing transcriptional similarities between samples. Dissimilarity scores were calculated from a

normalised abundance matrix of genes differentially expressed between E13.5 XY and E13.5 XX gonads. Blue boxes indicate the similarity between induced Sertoli-like cells (iSC-2TF) and the E10.5-E13.5 *in vivo* gonadal cells. The iSC-2TF are more similar to male-derived gonadal cells (black arrows) than to female derived gonadal cells (white arrows). Relative sample dissimilarity is shown running from dark purple (dissimilar) to yellow (similar).

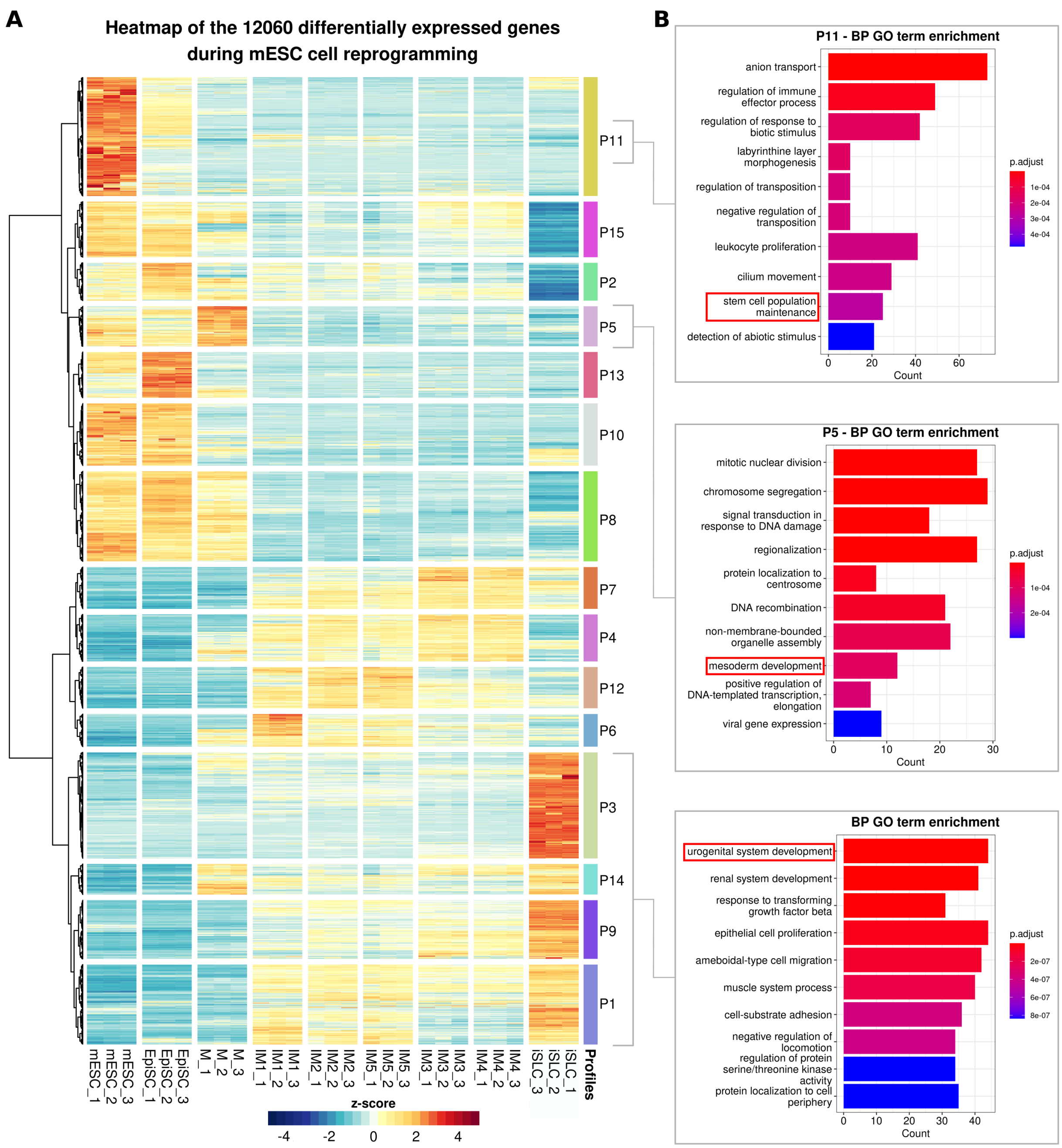

Supplementary Figure 7. Characterising the transcriptome changes during the *in vitro* differentiation. (A) heatmap representing the expression profiles of the genes significantly differentially expressed along cell reprogramming. Gene changes were highlighted by calculating the z-score. A low score means the gene expression is lower than the average expression, a high score at the contrary means a higher expression than the average, and a score of zero shows expression equal to the average. Genes were classified in 15 expression profiles (P1 to P15) using hierarchical clustering. (B) GO term analysis of the genes overexpressed in the mESCs (P11), M (P5) and the iSLC (P1-3-9-14).

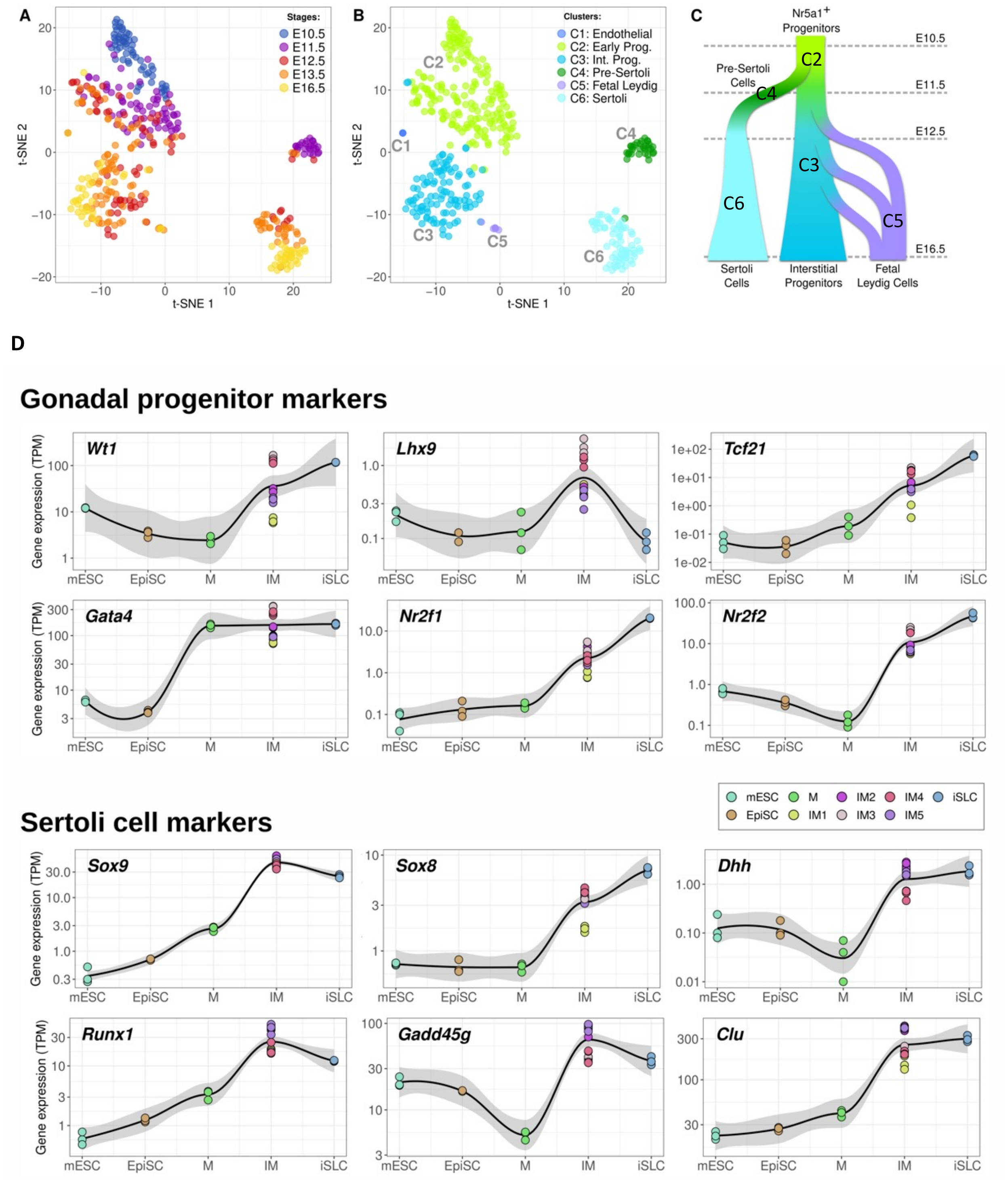

Supplementary Figure 8. Comparison of the bulk RNA-seq data to scRNA-seq gonadal data and marker analysis. (A-C) Analysis of the 435 NR5A1-eGFP sorted cells from XY mouse embryos from five different developmental stages (E10.5, E11.5, E12.5, E13.5, and E16.5) from Stévant et al. *(15)*. (A and B) t- SNE visualisation of the cell clustering, coloured by embryonic stages (A) and by cell clusters (C1-C6) (B). (C) Schematic representation of the NR5A1+ cell

lineage during fetal testicular development and the identity of the C2-C6 clusters. (Adapted from Stévant et al. *(15)*). (D) Gene expression analysis (normalised read counts) based on the bulk RNA-Seq of the *in vitro* differentiated cells on selected markers of gonad progenitors (The C2 cluster) (*Gata4*, *Lhx9*, *Wt1*, *Tcf21/Pod1*, *Nr2f1,* and *Nr2f2*) and Sertoli cells (The C4/C6 clusters) (*Sox9*, *Gadd45g*, *Sox8*, *Mro*, *Runx1,* and *Dhh*).

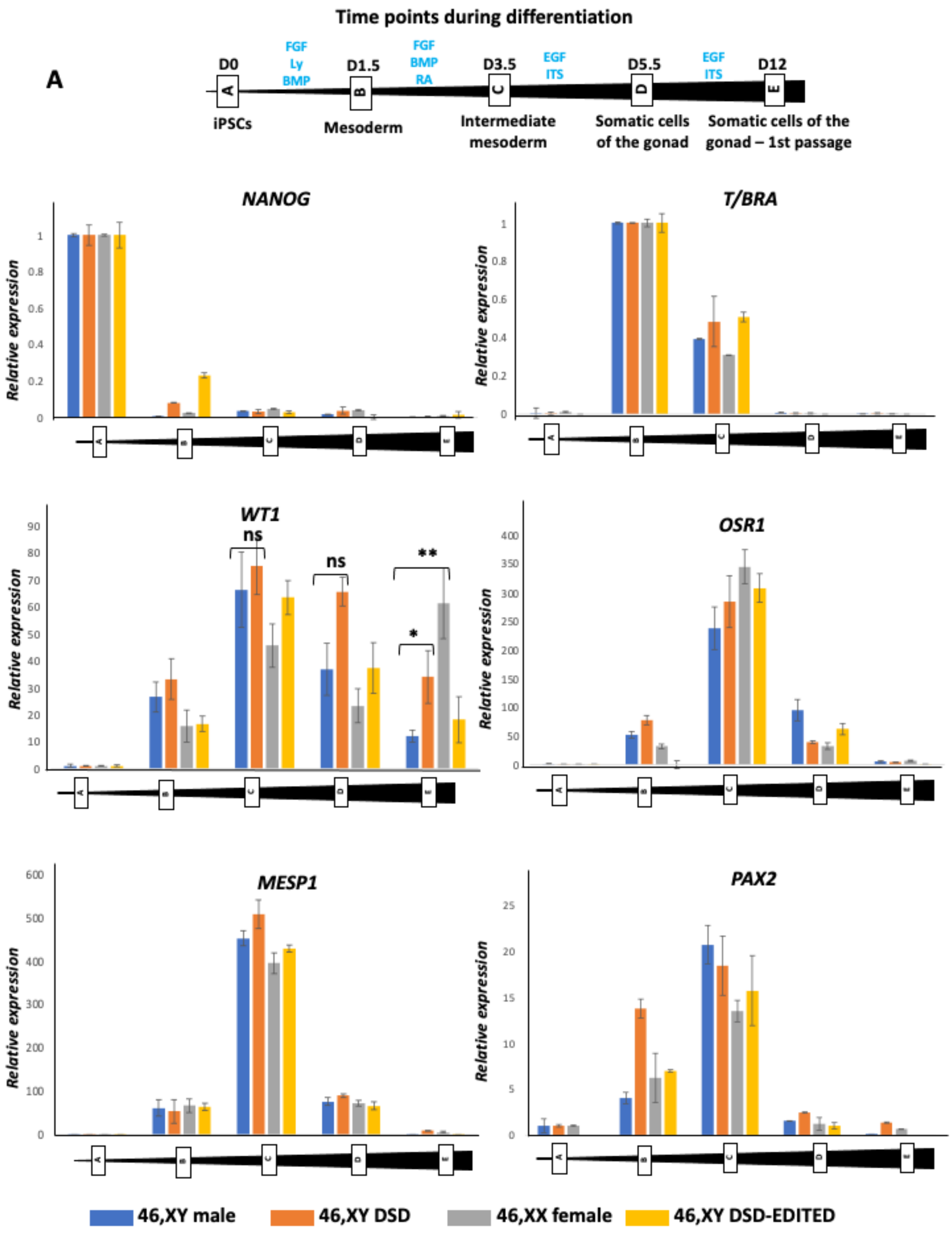
A
Time points during differentiation
D0
FGF
Ly
BMP
D1.5
FGF
BMP
RA
D3.5
EGF
ITS
D5.5
EGF
ITS
D12
iPSCs
Mesoderm
Intermediate mesoderm
Somatic cells of the gonad
Somatic cells of the gonad – 1st passage
NANOG
T/BRA
WT1
OSR1
MESP1
PAX2
Relative expression
ns
ns
**
*
46,XY male
46,XY DSD
46,XX female
46,XY DSD-EDITED

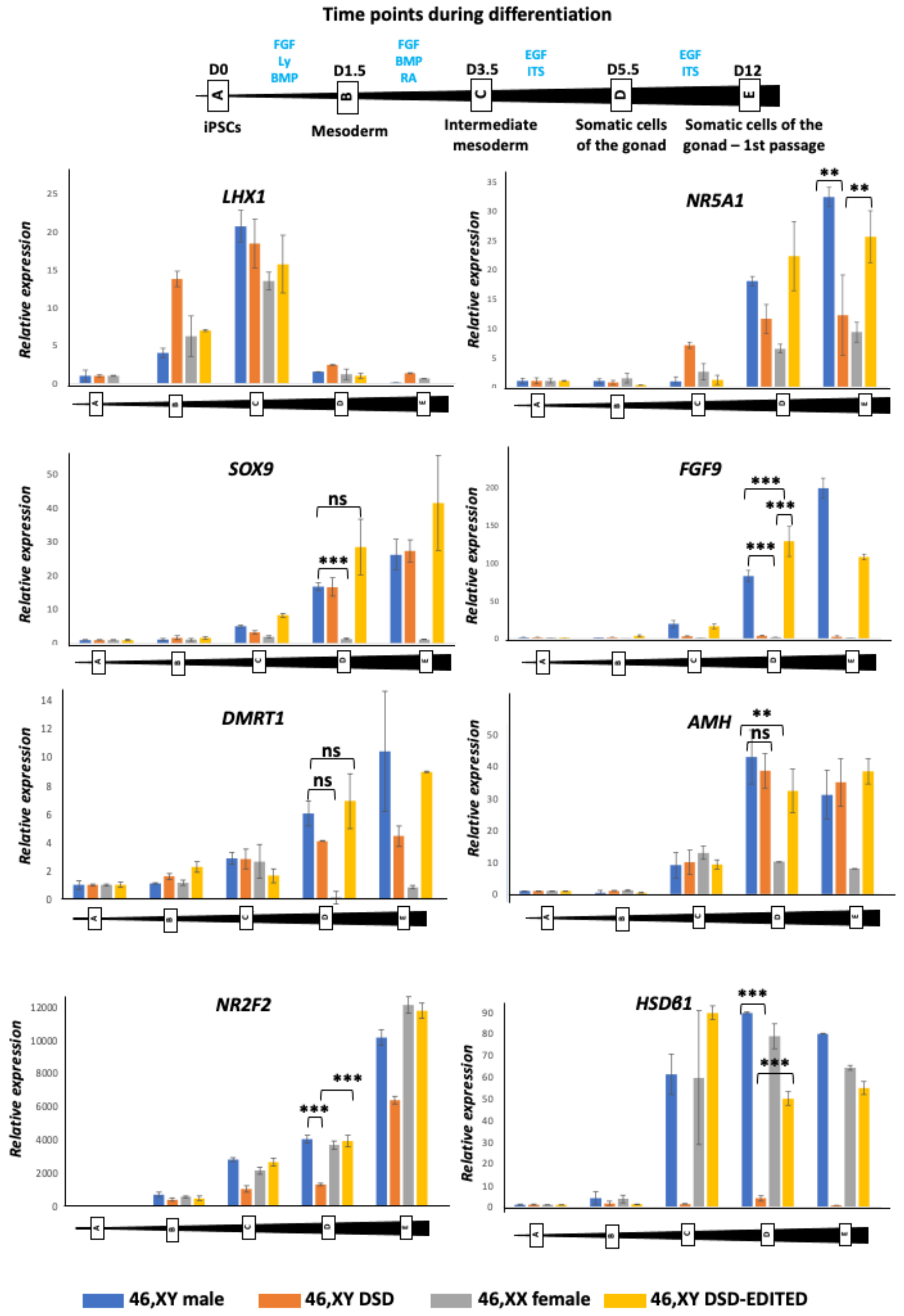
Time points during differentiation
FGF
Ly
BMP
FGF
BMP
RA
EGF
ITS
EGF
ITS
D0
D1.5
D3.5
D5.5
D12
A
B
C
D
E
iPSCs
Mesoderm
Intermediate mesoderm
Somatic cells of the gonad
Somatic cells of the gonad – 1st passage
LHX1
NR5A1
SOX9
FGF9
DMRT1
AMH
NR2F2
HSDβ1
Relative expression
**
***
ns
46,XY male
46,XY DSD
46,XX female
46,XY DSD-EDITED

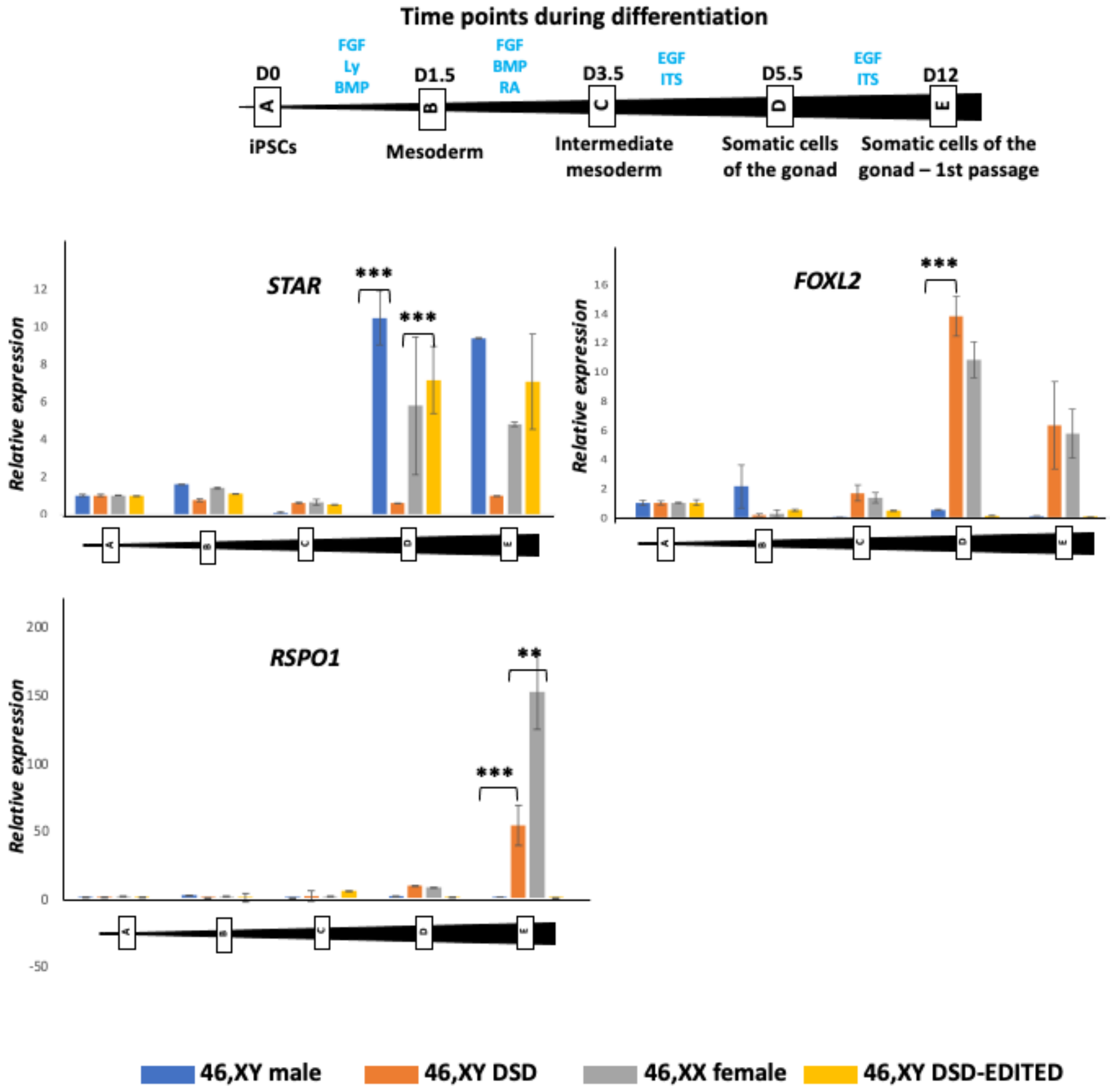

Supplementary Figure 9. Representative RT-qPCR expression profiles. Representative RT-qPCR expression profiles of selected differentiation markers of the four iPSC-derived cell lines (derived from healthy 46,XY male, 46,XX female, a 46,XY DSD patient with a pathogenic variant in *NR5A1* and the NR5A1 CRISPR/CAS9 corrected 46,XY DSD cells (46,XY DSD-EDITED)) during the differentiation process. Statistical analyses were carried out using GraphPad Prism 9 software (GraphPad). Quantitative data were subjected to a one-way ANOVA (* < 0.05, ** < 0.01, *** < 0.001, ns= not significant) followed by Bonferroni comparison.

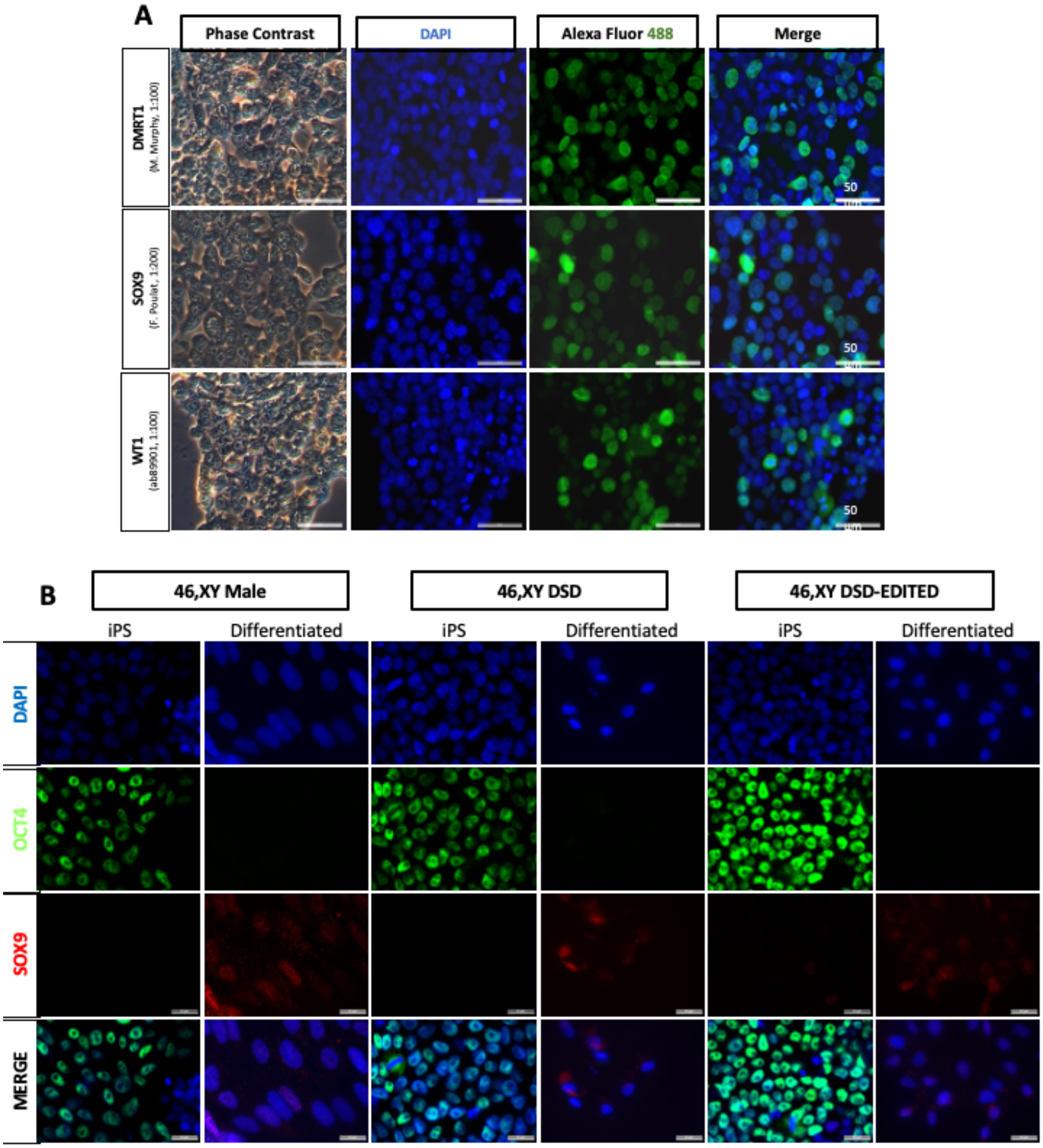

A
Phase Contrast
DAPI
Alexa Fluor 488
Merge
DMRT1
(M. Murphy, 1:100)
SOX9
(F. Poulat, 1:200)
WT1
(ab89901, 1:100)
50
B
46,XY Male
46,XY DSD
46,XY DSD-EDITED
iPS
Differentiated
DAPI
OCT4
SOX9
MERGE

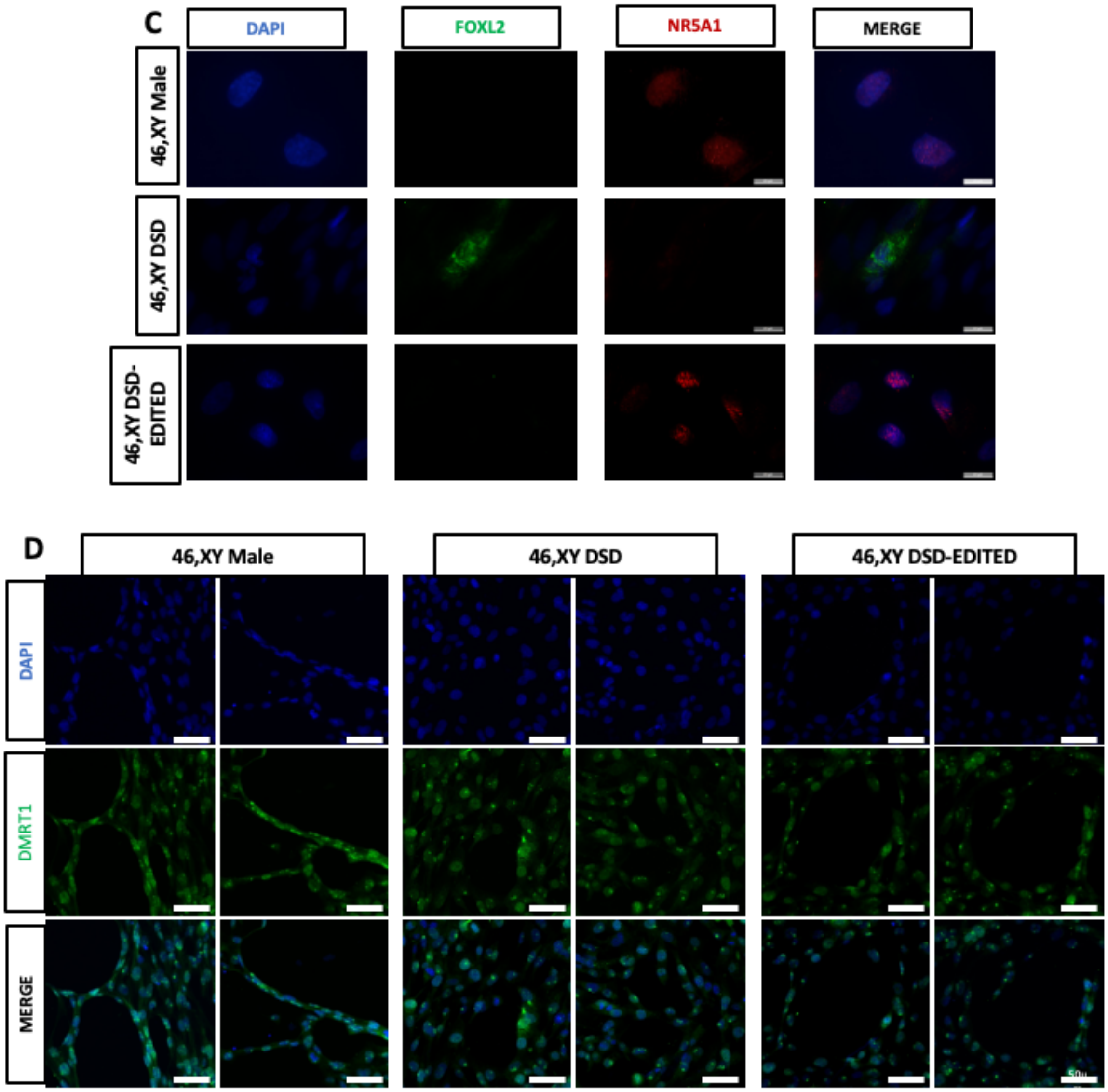
C
DAPI
FOXL2
NR5A1
MERGE
46,XY Male
46,XY DSD
46,XY DSD-EDITED
D
46,XY Male
46,XY DSD
46,XY DSD-EDITED
DAPI
DMRT1
MERGE

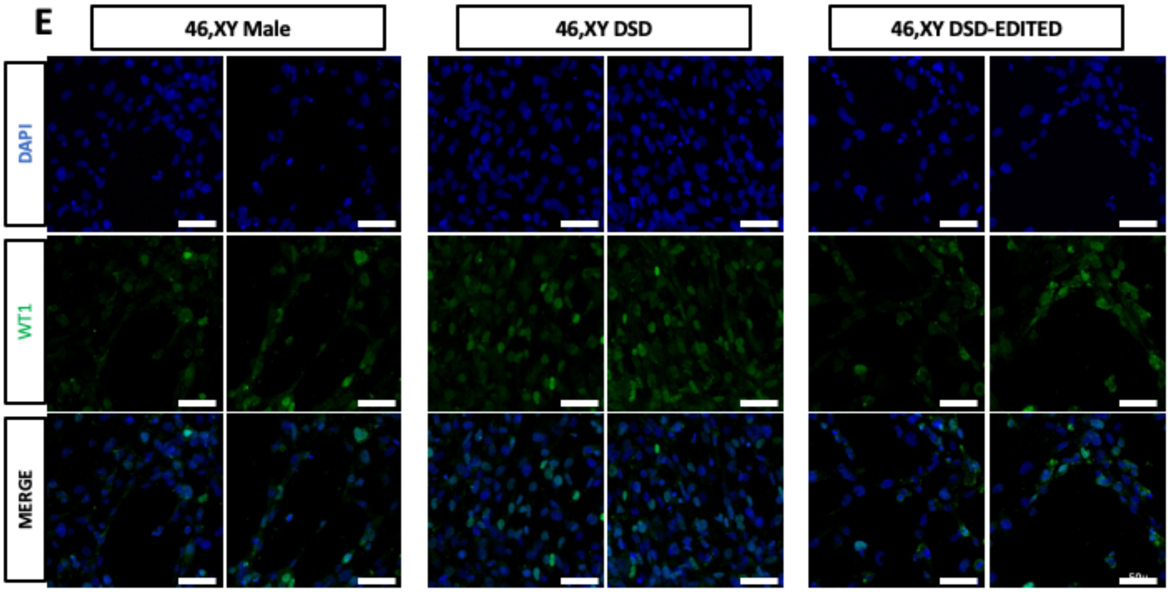

Supplementary Figure 10. Immunocytochemistry. (A) validation of antibody specificity on HEK 293-T cells transfected with *SOX9*, *DMRT1*, or *WT1* expressing plasmid (bar =50 µm); (B) OCT4 expression in hiPSCs and somatic cells of the gonad derived from 46,XY healthy male, 46,XY DSD and 46,XY DSD-EDITED hiPSCs (bar =20 µm); (C) NR5A1 and FOXL2 expression in somatic cells of the gonad derived from 46,XY healthy male, 46,XY DSD and 46,XY DSD-EDITED hiPSCs (bar =20 µm) and (D) DMRT1 and (E) WT1 expression in somatic cells of the gonad derived from 46,XY healthy male, 46,XY DSD and 46,XY DSD-EDITED hiPSCs that have been culture for over 3 months (bar =20 µm).

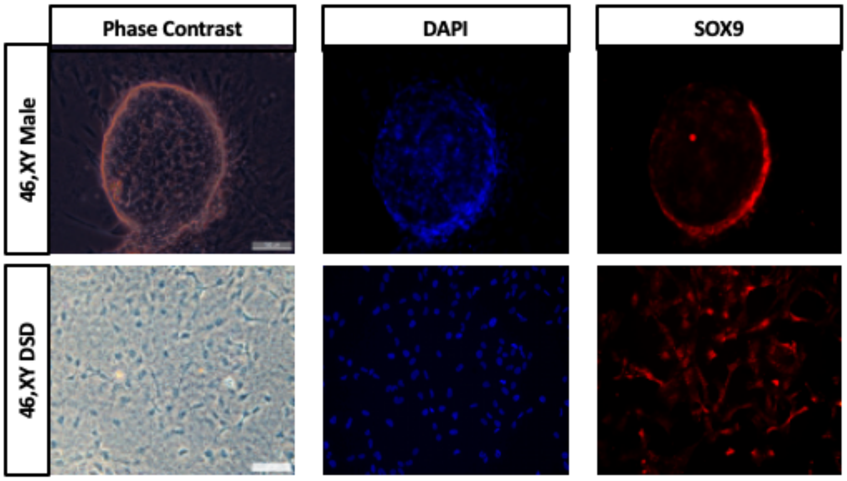

Supplementary Figure 11. Spontaneous tubular structure formation. Phase contrast of iPSC-derived from 46,XY and 46,XY DSD cells after 12 days of sequential culture in conditioned media, shows spontaneous formation of tubular structures in 46,XY that are composed of SOX9 positive cells. The cells derived from 46,XY DSD express SOX9 after 12 days of sequential culture in conditioned media but do not show defined structures.

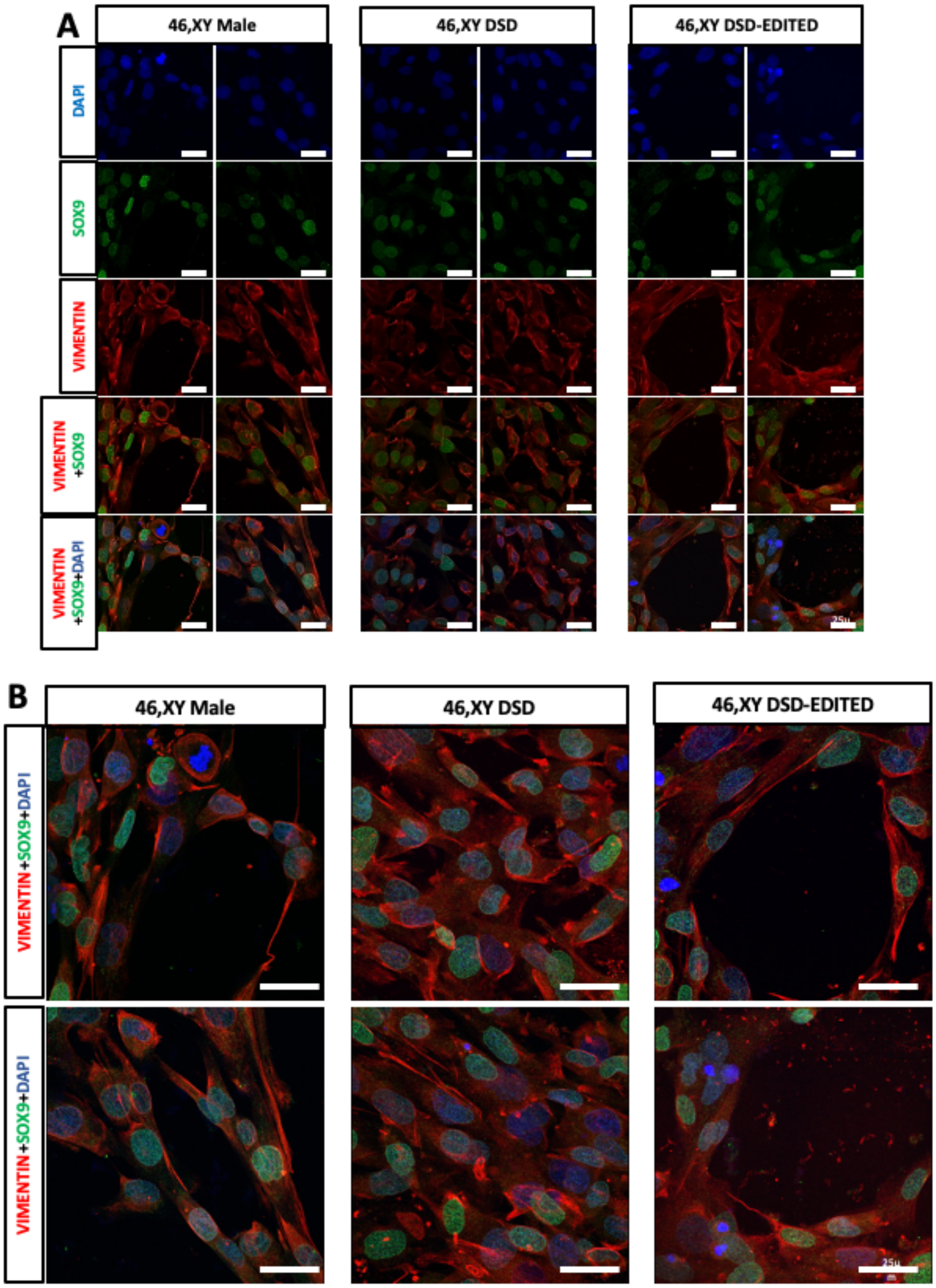

Supplementary Figure 12. Immunocytochemistry. SOX9 and VIMENTIN expression in somatic cells of the gonad derived from 46,XY healthy male, 46,XY DSD and 46,XY DSD-EDITED hiPSCs that have been culture for over 3 months (bar =50 µm (A) and bar =25 µm (B)). (B) Corresponds to still images from the 3D images (Supplementary movies 5-7).

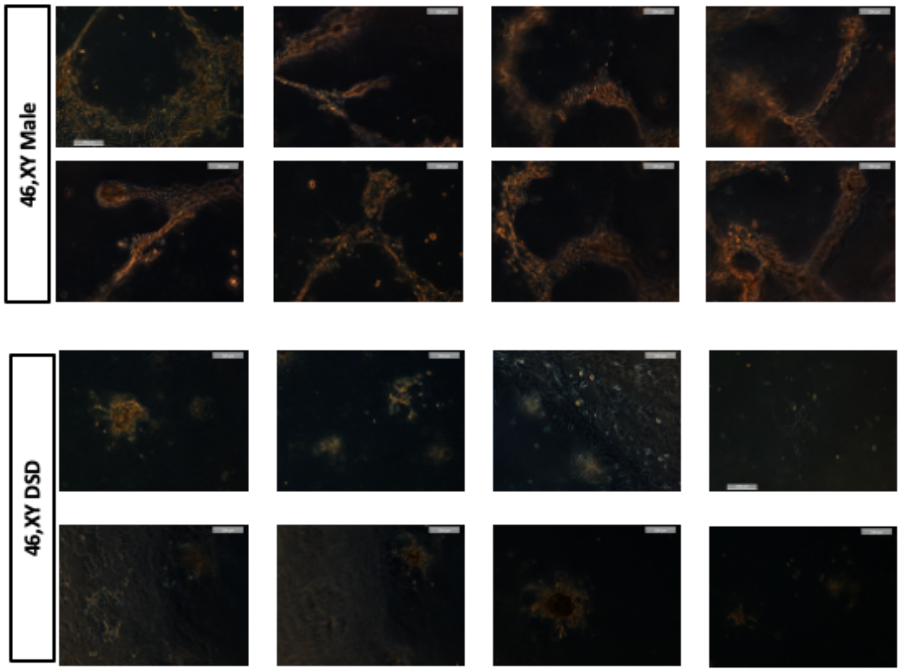

Supplementary Figure 13. Tubular structure formation. After FACS sorting, the iPSC-derived CLAUDIN11-positive cells from 46,XY male, 46,XY DSD cells were expanded for 7-14 days in Sertoli cell medium. These cells were then seeded on a constrained space on hardened (50%) Matrigel domes. The iPSC-derived cells from the 46 XY male migrate and form tubular structures whereas, 46,XY DSD cells form aggregates.

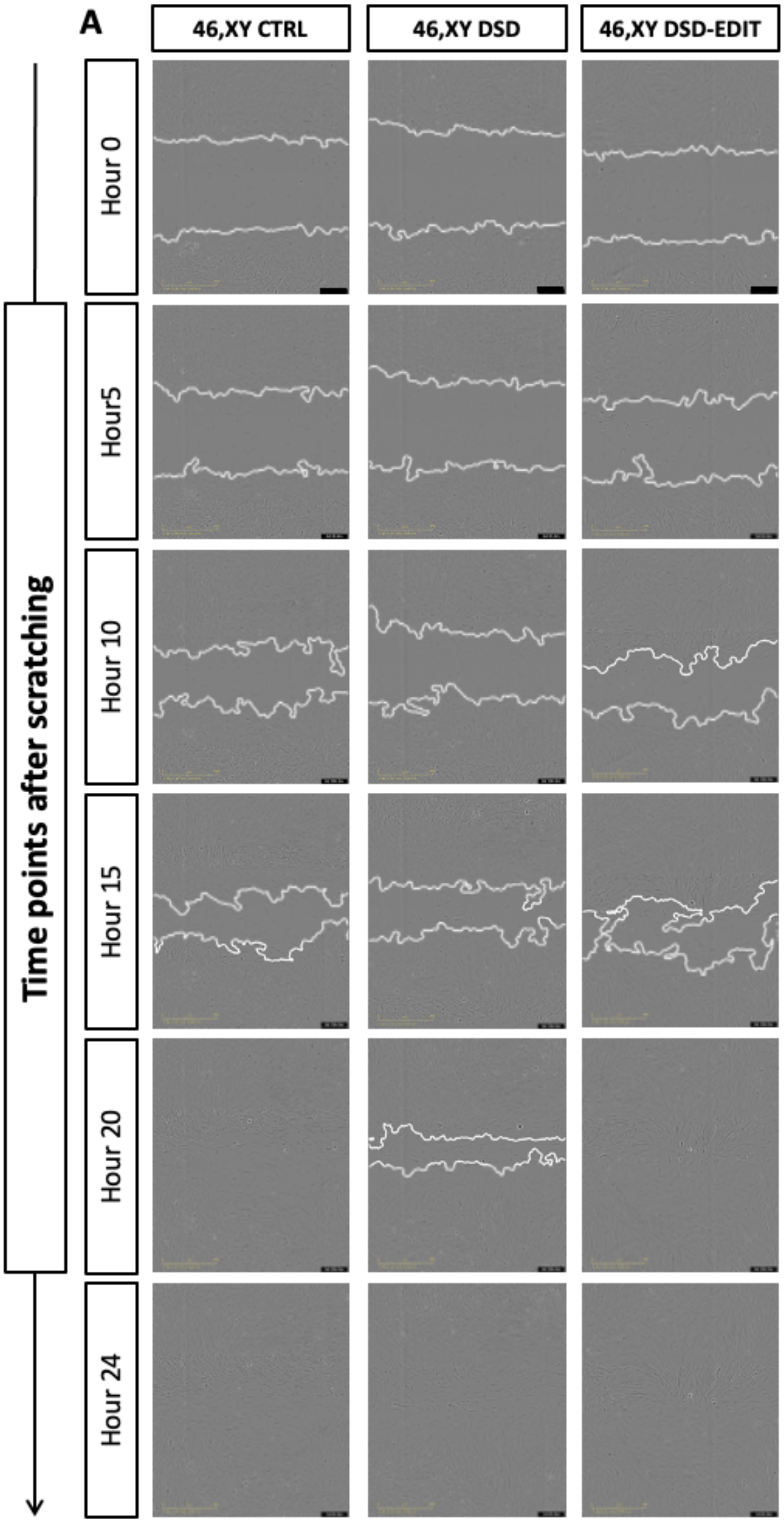

A
46,XY CTRL
46,XY DSD
46,XY DSD-EDIT
Time points after scratching
Hour 0
Hour5
Hour 10
Hour 15
Hour 20
Hour 24

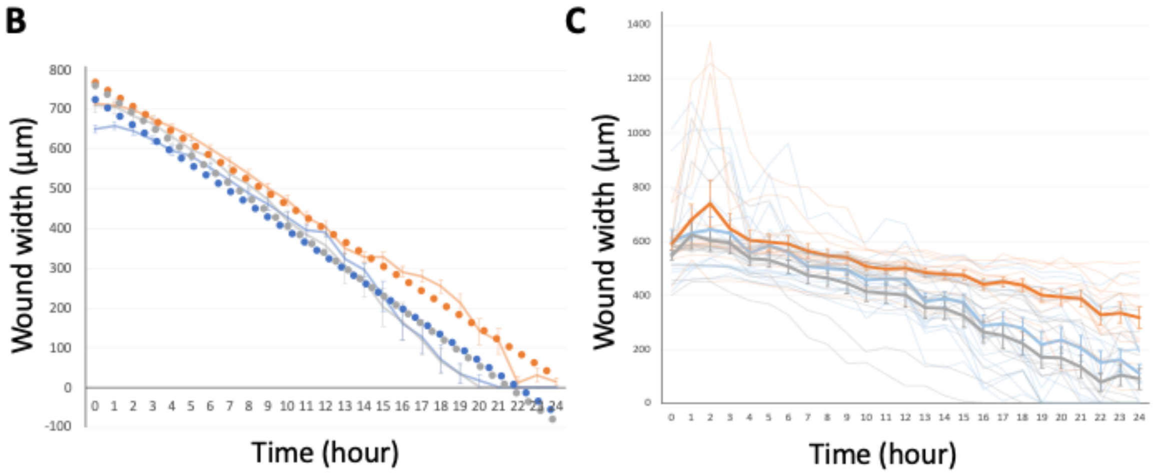

	p
**CTRL vs DSD**	**0,017**
**EDIT vs DSD**	**5,26 10^{-7}**
CTRL vs EDIT	0,063

	Wilcoxon *p*
**CTRL vs DSD**	**0,032**
**EDIT vs DSD**	**0,0055**
CTRL vs EDIT	0,9076

46,XY male **46,XY DSD** **46,XY DSD-EDITED**

Supplementary Figure 14. Scratch wound healing assay. (A) Representative images of wound healing at defined time points for somatic cells of the gonad derived from 46,XY, 46,XY DSD and 46,XY DSD-EDITED cells. The wound edges were calculated using the Incucyte® Scratch Wound Analysis Software Module (Sartorius Cat. No. 9600-0012) and shown in white (bars = 200 µm). The movement and velocity of the cells was plotted onto graph and the significance of difference between the slopes was calculated using (B) an online statistics calculator (http://www.danielsoper.com/statcalc) and (C) Wilcoxon Signed-Rank Test Calculator (https://www.aatbio.com/tools/mann-whitney-wilcoxon-signed-rank-test-calculator).

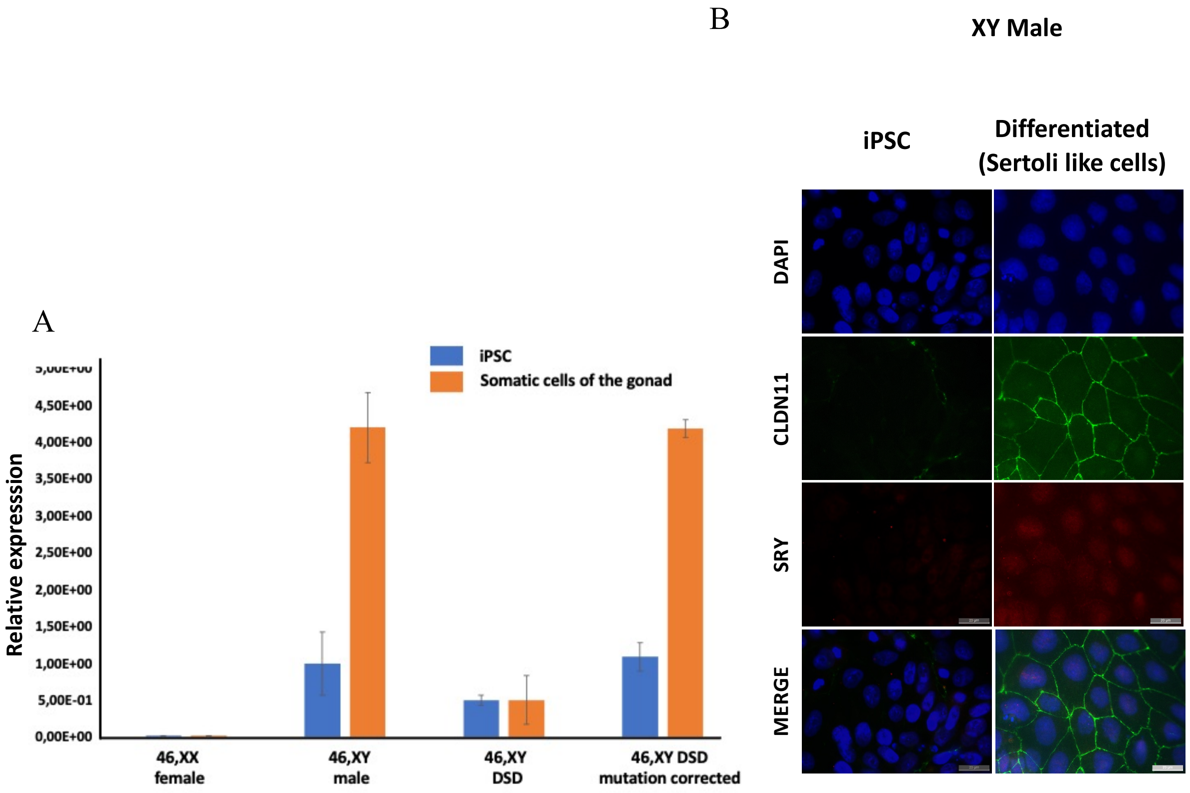

Supplementary Figure 15. SRY expression during human iPSC differentiation. (A) Representative RT-qPCR expression profile of *SRY* in the four iPSC cell lines and the somatic cells of the gonad derived from these cell lines at the end of the differentiation process. (B) Immunocytochemistry for SRY and CLAUDIN11expression in Sertoli-like cells derived from hiPSC-derived cells from a 46,XY healthy male (bar = 20 μ).

Table S1: Primers for genotyping mice

**Table S1. Primers for genotyping mice**			
**Primer name**	**Description**	**Sequence 5' to 3'**	**Product and size**
CFP-F	CFP	CGTGACCACCCTGACCTGG	F+R: 400 bp product
CFP-R	CFP	GTGGCGGATCTTGAAGTTGG	
oIMR8052	R26-M2rtTA_R_Mutant_SA site	GCGAAGAGTTTGTCCTCAACC	All 3 together: Mutant = 300 bp
oIMR8545	R26-M2rtTA_F_R26_Common	GAAAGTCGCTCTGAGTTGTTAT	Heterozygote = 300 bp and 603 bp
oIMR8546	R26-M2rtTA_R_R26	GGAGCGGGAGAAATGGATATG	Wild type = 603 bp
Sex-F	X/Y chromosome	GATGATTTGAGTGGAAATGTGAGGTA	McFarlane et al., 2013
Sex-R	X/Y chromosome	CTTATGTTTATAGGCATGCACCATGTA	

Table S2: Primers for RT-qPCR

**Table S2. Primers for Real-time quantitative RT-PCR**			
**Primer name**	**Gene**	**Marker of**	**Sequence 5' to 3'**
mKLF4_F	*Klf4*	*ESC*	GACTAACCGTTGGCGTGA
mKLF4_R			GCCACTCTCCAGGTCTGTG
mFGF5_F	*Fgf5*	*EpiSC*	ACTCCATGCAAGTGCCAAAT
mFGF5_R			TCGGCCTGTCTTTTCAGTTC
mBra_F	*Bra (T)*	*Mesoderm*	AAGAACGGCAGGAGGATGT
mBra_R			TCACGAAGTCCAGCAAGAAA
mTbx6_F	*Tbx6*	*Mesoderm*	ACCGCTACCCTGATTTGGATA
mTbx6_R			AGATGGGAGAAGGGGCAAAG
mLhx1_F	*Lhx1*	*Intermediate mesoderm*	CCCATCCTGGACCGTTTCC
mLhx1_R			CGCTTGGAGAGATGCCCTG
mOsr1_F	*Osr1*	*Intermediate mesoderm*	GACCGCGGCGGAACAAGATA
mOsr1_R			CACTGTGGGCAGGCCATTCA
mPax2_F	*Pax2*	*Intermediate mesoderm*	AAGCCCGGAGTGATTGGTG
mPax2_R			CAGGCGAACATAGTCGGGTT
mWt1_F	*Wt1*	*Intermediate mesoderm/ Genital ridge*	TTGAATGCATGACCTGGAATCA
mWt1_R			TTCCCTTTAAGGTAGCTCCTAGGTT
mLhx9_F	*Lhx9*	*Genital ridge*	ACCAGCAGCCTTATCCACCTTCACAG
mLhx9_R			TGTAATGCCCCAAGATTTGTTCTCCC
mCbx2_F	*Cbx2/M33*	*Genital ridge*	GGCTGGTCCTCCAAACACAA
mCbx2_R			CCCTGGGTCTCTTGCCTCT
mPod1_F	*Pod1*	*Genital ridge*	CTCCCTGAAAGTGGACTCCAA
mPod1_R			CGGGCTTTTCTTAGTGGGC
mFog2_F	*Fog2*	*Genital ridge*	ACCAGGAGAGCTAGAAGTGTTT
mFog2_R			GGACCTGAGCCTTCGTCTT
mGata4_F	*Gata4*	*Genital ridge/ Sertoli cells*	CCCCAATCTCGATATGTTTGATG
mGata4_R			TTGACACACTCTCTGCCTTCTGA
mSox9_F	*Sox9*	*Sertoli cells*	AAGAAAGACCACCCCGATTACA
mSox9_R			CAGCGCCTTGAAGATAGCATT
mSf1_F	*Nr5a1/Sf1*	*Genital ridge/ Sertoli cells*	CCTCGATGTGAAATTCCTGAACA
mSf1_R			TCCTGGGCGTCCTTTACG
mDmrt1_F	*Dmrt1*	*Sertoli and Germ cells*	GGAGTCTCCCAGCACCTTACG
mDmrt1_R			TCTGCCACTGGTTTCCAGTCT
mPBGD_F	*PBGD*	*HK gene*	CCTGGCATACAGTTTGAAATCAT
mPBGD_R			TTTTTCCAGGGCGTTTTCT

Table S3. Antibody list				
**Protein**	**Cat. No.**	**Raised in**	**Company**	**Dilution**
**Mouse ESC experiments**				
BRACHURY	AF2085	Goat	R&D Systems	1/150
WT1	ab89901	Rabbit	Abcam	1/400
GATA4	sc-25310	Mouse	Santa Cruz Biotechnology	1/300
SOX9	AF3075	Goat	R&D Systems	1/200
3β-HSD	sc-515120	Mouse	Santa Cruz Biotechnology	1/200
α-SMA	A2574	Mouse	Sigma Aldrich	1/400
DDX4	ab13840	Rabbit	Abcam	1/300
**Human ESC experiments**				
SOX9	14-9765-82	Mouse	ThermoFisher Scientific	1/100
OCT4	ab19857	Rabbit	Abcam	1/100
FOXL2	ab5096	Goat	Abcam	1/100
CLAUDIN11	36-4500	Rabbit	Life Technologies	1/100
DMRT1	/	Rabbit	Gift from Prof. David Zarkower	1/100
WT1	ab89901	Rabbit	Abcam	1/100
VIMENTIN	MA5-11883	Mouse	ThermoFisher Scientific	1/200
SRY	ab140309	Mouse	Abcam	1/100

Table S3: Antibody List

Other Supplementary Materials for this manuscript include the following:

Table S4: Measurement of wound closure measured by calculating the decrease in the wound width over time.

Supp data S1. Selected Gene marker list used in Figure S2D

Supp data S2. DE_genes_Gonen_LRT

Supp data S3. DE_gene_clusters

Supp data S4. P11_GO_terms

Supp data S5. P5_GO_terms

Supp data S6. iSLC (P1-3-9-14)_GO_terms

Supp data S7. XY_gonadal_marker_genes_Stevant_et_al_2018

Movie S1. supplementary video 1- 46,XY male

Movie S2. supplementary video 2- 46,XX female

Movie S3. supplementary video 3- 46,XY DSD

Movie S4. supplementary video 4- 46,XY DSD- EDITED

Movie S5. Supplementary video 5- 46,XY male

Movie S6. Supplementary video 6- 46,XY DSD

Movie S7. Supplementary video 7- 46,XY DSD-EDITED
